# A C^∧^S-Cyclometallated Gold(III) Complex as a Novel Antibacterial Candidate Against Drug-Resistant Bacteria

**DOI:** 10.3389/fmicb.2022.815622

**Published:** 2022-03-03

**Authors:** Carlos Ratia, Virginio Cepas, Raquel Soengas, Yolanda Navarro, María Velasco-de Andrés, María José Iglesias, Francisco Lozano, Fernando López-Ortiz, Sara M. Soto

**Affiliations:** ^1^ISGlobal, Hospital Clínic, Universitat de Barcelona, Barcelona, Spain; ^2^Área de Química Orgánica, Centro de Investigación CIAIMBITAL, Universidad de Almería, Almería, Spain; ^3^Immunoreceptors del Sistema Innat i Adaptatiu, Institut d’Investigacions Biomèdiques August Pi i Sunyer, Barcelona, Spain; ^4^Servei d’Immunologia, Centre de Diagnòstic Biomèdic, Hospital Clínic de Barcelona, Barcelona, Spain; ^5^Departament de Biomedicina, Universitat de Barcelona, Barcelona, Spain

**Keywords:** gold(III) antimicrobial, cycloaurate, MDR, MRSA, synergy

## Abstract

The worldwide emergence and spread of infections caused by multidrug-resistant bacteria endangers the efficacy of current antibiotics in the clinical setting. The lack of new antibiotics in the pipeline points to the need of developing new strategies. Recently, gold-based drugs are being repurposed for antibacterial applications. Among them, gold(III) complexes have received increasing attention as metal-based anticancer agents. However, reports on their antibacterial activity are scarce due to stability issues. The present work demonstrates the antibacterial activity of the gold(III) complex **2** stabilized as C^∧^S-cycloaurated containing a diphenylphosphinothioic amide moiety, showing minimum inhibitory concentration (MIC) values that ranged from 4 to 8 and from 16 to 32 mg/L among Gram-positive and Gram-negative multidrug-resistant (MDR) pathogens, respectively. Complex **2** has a biofilm inhibitory activity of only two to four times than its MIC. We also describe for the first time a potent antibacterial synergistic effect of a gold(III) complex combined with colistin, showing a bactericidal effect in less than 2 h; confirming the role of the outer membrane as a permeability barrier. Complex **2** shows a low rate of internalization in *Staphylococcus aureus* and *Acinetobacter baumannii*; it does not interact with replication enzymes or efflux pumps, causes ultrastructural damages in both membrane and cytoplasmic levels, and permeabilizes the bacterial membrane. Unlike control antibiotics, complex **2** did not generate resistant mutants in 30-day sequential cultures. We detected lower cytotoxicity in a non-tumoral THLE-2 cell line (IC_50_ = 25.5 μM) and no acute toxicity signs *in vivo* after an i.v. 1-mg/kg dose. The characterization presented here reassures the potential of complex **2** as a new chemical class of antimicrobial agents.

## Introduction

Among the many challenges to health, infectious diseases stand out for their ability to have a deep impact on humans and, therefore, have become a public health priority worldwide. Yet, the treatment of such infectious diseases remains a significant and global threat due to the emerging multidrug-resistant (MDR) or even pan-resistant pathogens refractory to virtually all clinically used antibiotics (Boucher et al., 2009). According to the Centers for Diseases Control and Prevention (CDC), 2.8 million antibiotic-resistant infections were reported in the United States in 2019, leading to more than 35,000 deaths (CDC, 2019). In Europe, it is estimated that more than 650,000 people suffer from infections caused by MDR bacteria each year, and 33,000 people die as a result. In February 2017, the World Health Organization (WHO) published a list of antibiotic-resistant priority pathogens that pose the greatest threat to human health. The panel of experts heartily recommended that future R + D strategies should be focused on the discovery and development of new antibiotics specifically active against MDR Gram-negative bacteria (Shrivastava et al., 2018).

Since 2017, only two new antibiotics contain a new active substance (vaborbactam–meropenem and lefamulin), and the remaining pipeline consists mostly of reformulations of already existing antibiotics with conventional targets (Theuretzbacher et al., 2020; World Health Organization [WHO], 2020). Bacteria not only exist as planktonic cells but they can adhere to surfaces and form structured communities, commonly named biofilms. Biofilm-mediated infections are frequently untreatable, thus, being of great medical concern. Even if several synthetic and natural compounds have shown antibiofilm activity against resistant bacterial strains, the arsenal of antibiofilm agents is still very limited (Davies, 2003; Batoni et al., 2016). This clearly points to the need of novel antibacterial compounds, preferably from new chemical classes with new mechanisms of action.

Metal-containing compounds are the focus of great interest as innovative antibacterial agents (Frei, 2020; Frei et al., 2020). The form of the metal (e.g., coordination or organometallic complexes, nanoparticles), the oxidation state, the coordination numbers, and the ligands offer practically endless options for fine tuning their biocidal properties (Claudel et al., 2020; Saidin et al., 2021). In this regard, gold-based compounds are attracting much interest as antimicrobial agents. However, the use of gold in medicinal preparations goes back many of thousands of years. Ancient cultures, such as those in India (Parimalam et al., 2021) and Egypt (Higby, 1982) used gold-based medicines to treat diseases, but the first reports of the use of gold to cure sickness were found in China and date back as far as 2500 BC (Huaizhi and Yuantao, 2001). In modern medicine, gold compounds started to get noticed precisely for their antibacterial properties, when Robert Koch discovered at the end of the 19th century that potassium dicyanidoaurate(I) effectively inhibited *Mycobacterium tuberculosis* growth in cultures (Benedek, 2004). In the 1920s of the last century, Jacques Forestier used gold(I) polymeric thiolates for the treatment of rheumatoid arthritis (Kean et al., 1985). However, the turning point was the work of Sutton, who developed the orally administrable Au(I)-based drug auranofin in 1986 (Sutton, 1986). Since then, interest in the use of gold complexes in medicinal chemistry has grown exponentially.

The emergence of MDR bacteria has led to research on repurposing existing clinical molecules as antimicrobials. Such an effort led to the discovery of the potent antibacterial activity of antirheumatic gold(I) complex auranofin (Roder and Thomson, 2015). Subsequently, a relatively large number of gold(I) complexes have been synthesized and evaluated for their antibacterial activity (Balfourier et al., 2020). Recently, it was reported that the exchange of sulfur-containing ligands in auranofin with *N*-heterocyclic carbene (NHC) ligands led to the formation of linear gold(I) complexes showing interesting biological profiles (Baker et al., 2005). In recent years, medicinal applications of gold(I)–NHC complexes focused mostly on their anticancer activity (Samanta et al., 2015; Mora et al., 2019; Patil et al., 2020; Behera et al., 2021; Guarra et al., 2021). In contrast, notably fewer contributions describing significant antimicrobial activities have been reported (Roymahapatra et al., 2012; Samanta et al., 2013; Büssing et al., 2021; Hussaini et al., 2021; Jakob et al., 2021).

Gold(III) complexes have been intensively investigated as potential antitumor agents due to their similarities with platinum(II) complexes (Rana et al., 2018; Radisavljević and Petrović, 2020; Tong et al., 2020). Only recently, the growing threat of MDR pathogens has drawn attention to the application of these compounds also as antimicrobials (Glišić and Djuran, 2014; Dominelli et al., 2018). Gold(III) complexes that are stable in the biological media are based primarily on cyclometallated derivatives bearing C^∧^N pincer-type ligands (Chakraborty et al., 2021) due to the increased stability toward thiol-containing cellular reducing agents provided by the presence of at least one gold(III)–carbon σ bond (Jurgens et al., 2018).

Antibiofilm applications of gold complexes have been even less investigated. In this regard, bis-imidazolium gold(I) complexes displayed effective inhibition and eradication of pathogenic biofilms (Samanta et al., 2013). Recently, Pintus et al. (2017) reported a heteroleptic cyclometallated gold(III) complex containing *C*-deprotonated 2-benzylpyridine and 1,2-dicyanoethene-1,2-dithiolate bidentate ligands, which inhibited *Staphylococcus aureus* biofilm formation up to a concentration of 3.125 μg/ml. The bacterial toxicity of gold(III) complexes is assigned to non-specific simultaneous mechanisms, which significantly hampers the emergence of antimicrobial resistance. Although the precise molecular mechanisms of antibacterial gold compounds remain unknown, there is evidence on them targeting thiol-containing proteins via ligand exchange because of the strong affinity of sulfur to gold ions. This process could affect many proteins, and their binding to gold(III) ions may prevent them from performing their function. In addition, the easy reduction of Au(III) to Au(I) may cause disruption of redox and metal homeostasis (Chandrangsu et al., 2017; Balfourier et al., 2020). Both mechanisms may lead to bacterial cell death. The nucleophilic displacement of bromide in gold(III) complexes by free cysteine residues of proteins has been established by electrospray ionization – mass spectrometry (ESI-MS) studies (Massai et al., 2020).

The use of antibiotics in combination with different natural or synthetic compounds has recently emerged as an alternative strategy to overcome the problem of bacterial resistance (Leite et al., 2016; Moussaoui and Alaoui, 2016). Several organic and inorganic compounds have been reported to enhance the antibacterial activity of commonly used antibiotics against many resistant bacteria. Among them, metallic ions and metallic nanoparticles have been particularly promising when used in combination with several antibiotics in different Gram-positive and Gram-negative strains (Hwang et al., 2012; Shabatina, 2018). In connection with those findings, recent reports suggest that a combination therapy of antibiotics and gold materials, such as gold ions, could display a synergistic effect (Rai et al., 2010; Nazari et al., 2012). The only report in which the conventional antibiotic norfloxacin is coordinated to gold(III) shows that this complex exhibited higher activity against *Pseudomonas aeruginosa* than the reference antibiotic due to a synergistic effect (Refat, 2007). Using a single agent to treat bacterial infections in the clinical setting appears to have become less effective and, therefore, combination therapy has been chosen as treatment in cases of complicated infections. In this sense, the coordination of gold complexes to antibiotics may (i) enhance the activity of the selected antimicrobial agents; (ii) prevent or delay the emergence of resistances; and (iii) decrease required doses, reducing both costs and the chances of toxic effects (Walsh and Wencewicz, 2014).

**FIGURE 1:**
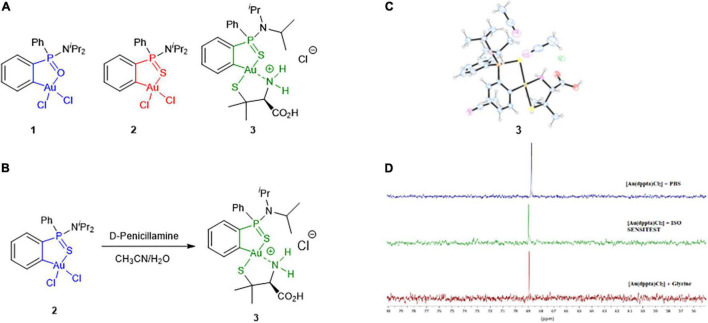
**(A)** Structures of the Au(III) complexes investigated in the study. **(B)** Synthesis of complex **3** including the numbering scheme used. **(C)** X-ray structure of complex **3**. **(D)** ^31^P NMR spectra of complex **2** in 5 M HCl in the presence of PBS (top), ISO-Sensitest broth (middle) and glycine (bottom) after 18 h of sample preparation.

We have previously reported the synthesis of phosphinic amide and phosphinothioic amide C^∧^X-cycloaurated complexes [Au(dppa)Cl_2_], **1** (X = O, dppa = diphenylphosphinic amide) (Oña-Burgos et al., 2009) and [Au(dppta)Cl_2_], **2** (X = S, dppta = diphenylphosphinothioic amide) (Belmonte-Sánchez et al., 2017) (Figure 1A). In view of the Au–halogen exchangeability in these complexes (Soengas et al., 2018) and the structural similarity with C^∧^N-cyclometallated gold(III) compounds showing antimicrobial properties (Glišić and Djuran, 2014; Jurgens et al., 2018; Chakraborty et al., 2021), we decided to investigate the activity of organometallic complexes **1** and **2** together with a new derivative **3** containing a penicillamine ligand against selected MDR bacteria. Subsequently, we evaluated in-depth the potential of the active complex **2** as a new antimicrobial agent and shed light on its bacterial mechanism of action by characterizing its (i) antibacterial and biofilm inhibitory activity against a wide panel of clinically relevant bacteria with a clear representation of pathogens listed on the WHO Priority List, (ii) synergy with current antimicrobials from different functional categories, (iii) bacterial intracellular uptake, (iv) interaction with DNA replication enzymes and efflux pumps, (v) ultrastructural changes in bacterial morphology after treatment, (vi) membrane permeabilization effect, (vii) ability to generate bacterial resistance, (viii) *in vitro* cytotoxicity in tumoral and non-tumoral cell lines, (ix) hemolytic activity, and (x) *in vivo* acute toxicity.

## Materials and Methods

### Chemistry

Reactions requiring an inert atmosphere were conducted under dry nitrogen, and the glassware was oven dried (120°C). All reagents were commercially available and were used without further purification. Nuclear magnetic resonance (NMR) spectra were obtained on a Bruker Avance III HD 300 (^1^H, 300.13 MHz; ^13^C, 75.47 MHz; and ^31^P, 121.49 MHz), Bruker Avance III HD 500 (^1^H, 500.13 MHz; ^13^C, 125.76 MHz; ^15^N, 50.68 MHz; and ^31^P, 202.46 MHz), and Bruker Avance III HD 600 (^1^H, 600.13 MHz; and ^31^P, 242.95 MHz). Chemical shifts are given in ppm using tetramethylsilane (TMS) as internal standard for ^1^H and ^13^C, and liquid NH_3_ for ^15^N and 85% H_3_PO_4_ for ^31^P as external standards. The following abbreviations are used to indicate the multiplicity of signals: s, singlet; d, doublet; t, triplet; q, quartet; and sep, septet. When two diastereoisomers are characterized together, many ^1^H and ^13^C signals appeared overlapped. To simplify the description of the NMR data, overlapped signals are indicated with a single label. Signals arising from different diastereoisomers are distinguished by a prime after the number of the atom. Infrared (IR) spectra were recorded using a Bruker Alpha FTIR instrument and registered in KBr pellets. High-resolution mass spectra (HRMS) were recorded on an Agilent Technologies LC/MSD-TOF and HP 1100 MSD spectrometer using electrospray ionization. Melting points were recorded on a Büchi B-540 capillary melting point apparatus and are uncorrected. UV-vis spectra were recorded on a JASCO V-530 polarimeter. Single crystal x-ray diffraction data were collected on a Bruker D8 Venture diffractometer at 100 K, using CuKα (λ = 1.54178 Å) radiation (see Supplementary Material for additional details). Complexes **1** (Oña-Burgos et al., 2009) and **2** (Belmonte-Sánchez et al., 2017) have been prepared following previously reported procedures. Their ^1^H and ^31^P-NMR spectra are in good agreement with those described in the literature. ^1^H NMR data of complex **2** in different solvents used in this study are included in the Supplementary Material. The procedure for the synthesis of complex **3** and its structural characterization are described in the Supplementary Material.

### Bacterial Strains, Growth Conditions, and Antibiotics

The activity of the gold(III) compound **2** was evaluated against clinically relevant isolates, selected based on their resistance phenotype, etiology, and biofilm production. The panel of microorganisms included MDR and/or biofilm-producing strains collected from the Hospital Clinic of Barcelona (Barcelona, Spain) of *P. aeruginosa*, methicillin-resistant/susceptible *Staphylococcus aureus* (MRSA/MSSA), *Staphylococcus epidermidis*, *Stenotrophomonas maltophilia*, *Haemophilus influenzae*, *Klebsiella pneumoniae*, *Acinetobacter baumannii*, and *Escherichia coli*. The panel also included eight *Salmonella* spp. (including five quinolone-resistant strains) and six *Burkholderia cepacia* complex strains, which were collected from the Hospital Universitario Central de Asturias (Oviedo, Spain) and Hospital Ramón y Cajal (Madrid, Spain).

Isolates were routinely grown on Columbia sheep blood agar (Becton Dickinson, Heidelberg, Germany) and incubated at 37°C for 18 h with some exceptions. *B. cepacia* complex strains were cultured for 48 h, and the *H. influenzae* isolates were grown in chocolate blood agar containing IsoVitaleX, at 37°C with 5% CO_2_. Reference strains *S. aureus* ATCC 25923, *S. aureus* ATCC 29213, *P. aeruginosa* ATCC 27853, *H. influenzae* ATCC 49247, *E. coli* ATCC 25922, *A. baumannii* ATCC 19606, and ATCC 17978 were added as controls. Matrix-assisted laser desorption ionization–time of flight (MALDI–TOF) mass spectrometry was used to confirm species identification.

Stock solutions for control antibiotics ciprofloxacin (CIP), tobramycin (TOB), colistin (CST), and daptomycin (DAP) were prepared according to manufacturer’s instructions. Experiments with DAP were carried out in media containing 50 μg/ml of Ca^2+^.

### Minimum Inhibitory Concentration Determination

The minimum inhibitory concentration (MIC) was determined by broth microdilution method following the Clinical and Laboratory Standards Institute (CLSI) guidelines (CLSI, 2020). Stock solutions of **2** were prepared in 100% dimethyl sulfoxide (DMSO, Sigma-Aldrich, Darmstadt, Germany) at 6,000 mg/L and stored at −20°C until use. First, serial two-fold dilutions of **2** ranging from 128 to 0.25 mg/L were performed in ISO-Sensitest broth or Haemophilus Test Medium (HTM) broth for *H. influenzae* isolates in 96-well round-bottom microtiter plates. After 18 h of incubation in agar plates, bacterial colonies were resuspended in 0.9% NaCl to reach a 0.5 McFarland (equivalent to 1.5 × 10^8^ colony forming unit (CFU)/ml) and then diluted to obtain a final concentration in the well of 5 × 10^5^ CFU/ml. In addition, control antibiotics CIP and TOB, and serial two-fold dilutions of DMSO at the same concentration as in the **2** assays were added as a control. The plates were incubated at 37°C for 18 h (with 5% CO_2_ for *H. influenzae* isolates) and were visually read for the absence of turbidity.

MIC values were defined as the lowest concentration of the compound that inhibited visible growth. All the experiments were carried out by triplicate.

### Biofilm Inhibitory Studies

The biofilm inhibitory activity of **2** was determined against the biofilm-forming strains included in the study. Serial two-fold dilutions of **2** ranging from 256 to 0.5 mg/L were performed in flat-bottomed 96-well microtiter plates, with the corresponding culture media to enhance biofilm formation. Luria Bertani (LB) broth supplemented with 0.25% glucose was used for *P. aeruginosa*, *K. pneumoniae*, and *A. baumannii* strains; M63 with 0.25% glucose for *E. coli*; TSB with 0.25% glucose for *S. aureus*, *S. epidermidis*, and *S. maltophilia*; Tryptic soy broth (TSB) diluted 1/20 for *Salmonella* spp., and supplemented brain–heart infusion (sBHI) broth for *H. influenzae* strains. Bacterial suspensions were prepared from overnight cultures, and the turbidity was adjusted to a 0.5 McFarland in the corresponding media for each species and consequently diluted to reach an inoculum of 5 × 10^6^ CFU/well. For *P. aeruginosa* and *B. cepacia* complex assays, biofilms were formed by immersing pegs of a modified polystyrene microtiter lid (Nunc TSP System, Nunc, Rockslide, Denmark). After 48 h incubation at 37°C in static, biofilms on microtiter plates or pegs were carefully rinsed three times with 1 × phosphate-buffered saline (PBS) to discard planktonic cells and exposed at 65°C until completely dry. Biofilms were then fully covered with a 1% crystal violet (CV) stain solution for 10 min at room temperature (Stepanović et al., 2007). The CV was then removed, washed with 1 × PBS to eliminate the excess of dye and heat-dried for 60 min. Biofilm formation was quantified by eluting the CV fixed to the biofilm in 33% glacial acetic acid, and absorbance of each well was measured at 580 nm using a microplate spectrophotometer (EPOCH, Biotek, Santa Clara, CA, United States).

The minimum biofilm inhibitory concentration (MBIC) was defined as the minimal concentration of the compound that led to a three-fold decrease in absorbance when compared with the growth control values. All the experiments were carried out in triplicate.

### Quantification of Gold(III) Complex **2** Bacterial Uptake

To determine the intracellular uptake of **2**, we quantified the Au present in bacterial lysates through inductively coupled plasma–mass-spectrometry (ICP-MS) after exposure to **2** at different timepoints in *S. aureus* and in *A. baumannii* as representatives for Gram-positive and Gram-negative bacteria, respectively. First, overnight cultures of MRSA 163501-000 and *A. baumannii* Cr17 were grown to reach an optical density (OD) of 0.6, then harvested and resuspended in ice-cold PBS to a 1 × 10^10^ CFU/ml cell density. Bacterial suspensions were allowed to equilibrate for 15 min at 37°C and treated right after with **2** (30 and 20 μg/ml for *S. aureus* and *A. baumannii*, respectively). Aliquots at 0, 5, and 8 min timepoints were immediately centrifuged to a bacterial pellet; washed three times in ice-cold PBS; plated for CFU determination; and frozen until lysis. Bacteria treated only with PBS and stock solution of **2** without bacteria were used as controls. Samples were digested with HNO_3_, H_2_O_2_, and HCl at 60°C and subsequently diluted in an HCl-thiourea solution. The presence of Au in the samples was detected through ICP-MS in an Agilent 7500ce equipment under standard conditions.

Results for the Au quantification on the bacterial lysates were analyzed, extrapolated to the **2** quantity, and expressed combined with the total amount of CFU to obtain the amount of **2** internalized by a single CFU (fg/CFU).

### Synergy Testing

The activity of **2** in combination with the antibiotics CST and CIP was evaluated by the microdilution two-dimensional checkerboard technique (Garcia and Isenberg, 2014). The setup of each assay plate evaluated two-fold dilutions of **2** with two-fold dilutions of the chosen antimicrobial, considering a concentration range that included, in the middle of the gradient, the MIC of each molecule against the tested strain. Inoculum size, culture media, and incubation conditions were the same as described above for the MIC microdilution method. Results are read at one point in time after 18 h by visual examination.

The fractional inhibitory concentration index (FICI) was calculated considering the following formula: FICI = FIC A + FIC B, where FIC A is the MIC of drug A in combination/MIC of drug A alone, and FIC B is the MIC of drug B in combination/MIC of drug B alone. Synergy was defined by an FICI ≤ 0.5. Interactions with FICI values ranging from 0.5 to 4 were classified as additive/indifferent. Antagonism was defined by an FICI ≥ 4 (Saiman, 2007).

### Time-Dependent Killing Assays

Synergistic results obtained from the checkerboard arrays were confirmed by time-dependent killing assays. Prior to inoculation, each tube of ISO-Sensitest broth was supplemented with **2** either alone or in combination with CST, in concentrations ranging from 0.03 × MIC to 2 × MIC for both antimicrobials. The tubes were inoculated to reach a bacterial density of 5 × 10^5^ CFU/ml per tube and incubated at 37°C with shaking. To determine the number of viable bacteria, aliquots were taken at specific time points (0, 2, 4, 8, 24, and 48 h), serially diluted 10-fold when needed, and plated onto LB agar. Plates were incubated at 37°C, and CFUs were enumerated after 18 h.

Synergy was defined as a ≥2 log decrease in viability (CFU/ml) between the combination and the most active agent alone. The combination had to show also a ≥2 log bacterial reduction compared with the starting inoculum.

### Gyrase and Topoisomerase IV Inhibition Assays

DNA decatenation and supercoiling enzymatic inhibition assays were performed using topoisomerase IV and gyrase kit by Inspiralis (Norwich, United Kingdom) for both *S. aureus* and *E. coli*. For topoisomerase IV assays, 200 ng of double-stranded kDNA was incubated with **2** and comparator antibiotic ciprofloxacin at different concentrations (0.5, 2.5, 5, 10, 15, and 25 mg/L) in the presence of *S. aureus* or *E. coli* Topo IV, in a final volume of 30 μl. Each test condition was incubated for 30 min at 37°C. Negative (DNA substrate with no enzyme or treatment) and positive (DNA substrate and enzyme without treatment) controls served as cutoff values for 100 and 0% inhibition, respectively. Reactions were stopped by adding 30 μl of STEB buffer (40% sucrose, 100 mM Tris–HCl pH 8, 1 mM EDTA, and 0.5 mg/ml of bromophenol blue) and 30 μl of chloroform/isoamyl alcohol (24:1). Enzymatic reactions were loaded in 1% agarose gels and stained with SYBR™ Safe (Invitrogen, Paisley, United Kingdom) to observe released minicircles, and bands were quantified with the Gene Tools software (Syngene, Cambridge, United Kingdom).

For gyrase supercoiling inhibition assays, 0.5 μg of relaxed pBR322 was incubated with **2** and ciprofloxacin in a range of concentrations (0.5, 2.5, 5, 10, 15, and 25 mg/L) in the presence of *S. aureus* or *E. coli* DNA gyrase, in a final volume of 30 μl. Each test condition was incubated for 30 min at 37°C. Negative (DNA substrate with no enzyme or treatment) and positive (DNA substrate and enzyme without treatment) controls served as cutoff values for 100 and 0% inhibition, respectively. Reactions were stopped as described above in decatenation assays. The two forms of the plasmid were observed by 1% gel electrophoresis, stained, and quantified in the same manner as for topoisomerase IV assays.

### Activity of Gold(III) Complex **2** in the Presence of Efflux Pump Inhibitors

To determine if the inhibition of efflux systems compromises the activity of **2**, the MIC of the compound in the presence of subinhibitory levels of efflux pump inhibitors (EPIs) was assessed against selected strains of *S. aureus*, *S. maltophilia*, *H. influenzae*, *P. aeruginosa*, and *E. coli*. Three EPIs were used in this study: phenylalanine-arginine β-naphthylamide (PaβN), carbonyl cyanide *m*-chlorophenyl-hydrazone (CCCP), and reserpine (RSP). Preliminary microdilution experiments against all strains were performed to define the fixed subinhibitory concentration of the EPIs that would be used in the study, which should be at least two-fold below their MIC. Consequently, each tested microorganism was exposed to PaβN at 16 mg/L, CCCP at various concentrations (0.25 mg/L for *S. aureus* and *H. influenzae*, 8 mg/L for *S. maltophilia* and *E. coli*, and 16 mg/L for *P. aeruginosa*), and RSP at 8 or 16 mg/L (for *S. aureus* and *P. aeruginosa* isolates) in the presence of **2** within a range of 0.25–128 mg/L. Culture conditions were as those described above for the broth microdilution assays. Plates were checked for fold variations in MIC between the activity of **2** alone or in the presence of any of the three EPIs tested.

### Transmission Electron Microscopy Analysis

Bacterial ultrastructural analysis after treatment with **2** was carried out by transmission electron microscopy (TEM) imaging. Exponential cultures of MRSA 163501-000, *P. aeruginosa* 953, and *A. baumannii* Cr17 were exposed to minimum inhibitory concentrations of **2** and were incubated for 30 min. These cells were consequently fixed with 2% glutaraldehyde and 4% paraformaldehyde, then postfixed for 2 h in 1% osmium tetroxide and 0.8% potassium ferrocyanide. Graded acetone series were used to dehydrate the samples prior to infiltrate an epoxy resin according to the Spurr’s formula. Ultrathin sections were cut with a diamond knife, picked up with Formvar-coated grids and counterstained with uranyl acetate and lead citrate. Samples were examined using a TEM JEOL J1010 microscope charged with a Gatan charge-coupled device (CCD) Orion camera with Digital Micrograph software, under standard operating conditions.

### Bacterial Membrane Permeabilization Assays

We evaluated the effect of complex **2** on membrane permeabilization against MRSA 163501-000 and *A. baumannii* Cr17 using the LIVE/DEAD Baclight™ viability kit (L13152, Molecular Probes, Life Technologies, Illkirch, France) following the manufacturer’s instructions. Bacterial cultures were harvested from logarithmic growth by centrifugation at 3,500 × *g* for 5 min, washed twice, and resuspended in saline to an OD_595_ = 0.5. Bacterial inoculum was then incubated with 6 μM SYTO9 and 30 μM of propidium iodide (PI) in the dark for 30 min. A total of 50 μl of SYTO9-PI-labeled cells was added to a 96-well plate and exposed to 50 μl of complex **2** serially diluted in saline, from 64 to 4 mg/L. Untreated controls were also included. The fluorescence intensity (SYTO9 = 480/500 nm excitation/emission; PI = 490/635 nm excitation/emission) was monitored using a Tecan microplate reader (Tecan, InfiniteM2000PRO, Zurich, Switzerland) for 30 min (in 5 min intervals) since the beginning of the exposure. Assays were carried out in triplicate and plotted as the mean ± SD PI fluorescence signal at each time point after subtracting the untreated control mean PI signal.

### Point of Resistance

We performed sequential culturing of *S. aureus* ATCC 29213 and *A. baumannii* ATCC 19606 in the presence of subinhibitory levels of **2** in order to increase the probability of obtaining resistant mutants. Bacterial cells were grown in 1 ml of media (ISO-Sensitest broth) containing **2** at different concentrations. DAP and CST were also included as a control for *S. aureus* and *A. baumannii*, respectively. Cells grown up to an OD = 1 were diluted 1:100 to complex **2** present at 0.25 × MIC, 0.5 × MIC, 1 × MIC, 2 × MIC, 4 × MIC, and 8 × MIC. At 24-h intervals, the cultures were checked for growth. Cultures from the highest concentration showing bacterial growth were diluted 1:100 into fresh media containing 0.25 × MIC, 0.5 × MIC, 1 × MIC, 2 × MIC, 4 × MIC, and 8 × MIC of **2**. This serial passaging was repeated daily for 30 days. Any culture that grew at higher than the MIC levels was passaged on drug-free ISO-Sensitest agar plates, and the MIC was then determined by broth microdilution.

### *In vitro* Cytotoxicity Assays

3-(4,5-dimethylthiazol-2-yl)-2,5-diphenyltetrazolium bromide (MTT)-based colorimetric assay measures the activity of cellular enzymes that reduce the tetrazolium dye, MTT, to insoluble formazan, giving a purple color. This assay measures mitochondrial metabolic activity via NAD(P)H-dependent cellular oxidoreductase enzymes and may, under defined conditions, reflect the number of viable cells.

The cells lines used in this study were HepG2 (HB-8065), a perpetual cell line, which was derived from the liver tissue of a 15-year-old Caucasian American male with a well-differentiated hepatocellular carcinoma; and THLE-2, an immortalized human liver cell (left lobe). THLE-2 cells express phenotypic characteristics of normal adult liver epithelial cells and constitute an *in vitro* model for pharmacotoxicological studies and drug toxicity.

Cells were seeded at a concentration of 1 × 10^4^ cells/well in 200 μl of Eagles’s minimum essential medium (MEM) and incubated at 37°C in 5% CO_2_. After 24 h, the medium was replaced with a final volume of 200 and 1 μl of compound (dilution 1/200), and controls were added to the plates. The curve of compound/extract was realized with 1/2 dilution in each step, 10 points by curve in acetonitrile (solvent of **2**). Methyl methanesulfonate (MMS, 8 mM) and DMSO (0.5%) were used as positive and negative control, respectively. The doxorubicin curve was used as internal control. Previously, a DMSO curve was tested with these cell lines, and no effects were observed with 1% DMSO. When compounds and controls were added, plates were incubated at 37°C in 5% CO_2_ incubator for 72 h. After this time, MTT solution was prepared at 5 mg/ml in 1 × PBS and then diluted at 0.5 mg/ml in MEM without phenol red. The sample solution in the wells was flicked off, and 100 μl of MTT dye was added to each well. The plates were gently shaken and incubated for 3 h at 37°C in 5% CO_2_ incubator. The supernatant was removed, and 100 μl of 100% DMSO was added. The plates were gently shaken to solubilize the formed formazan. The absorbance was measured using a multireader Victor™ at a wavelength of 570 nm. Molecule concentration ranged from 50 to 0.09 μM.

### Hemolytic Activity

Whole blood from the Banc de Sang i Teixits of Catalonia, according to the recommendation and approval of the Ethics Committee of Clinical Investigation from the Hospital Clinic of Barcelona, was centrifuged at 2,000 × *g* for 15 min to obtain separated erythrocytes. Cells were washed in 1 × PBS three times, and the hematocrit was adjusted to obtain a 1% final concentration in the wells. The suspension was exposed to increasing concentrations of **2** ranging from 150 to 0.09 μM in 96-well round-bottomed microtiter plates and incubated for 4 h at 37°C. Plates were centrifuged for 5 min at 1,000 rpm, and the absorbance of supernatants was measured at 540 nm to detect hemoglobin release. A value of 100% hemolysis was determined by incubating erythrocytes for 4 h at 37°C with 1% Triton X-100 (Knopik-Skrocka and Bielawski, 2005).

### *In vivo* Acute Toxicity

Female CD1 (ICR) mice (five mice/group) between 7 and 8 weeks of age were purchased from Charles River Laboratories (Écully, France). All the procedures were approved by the Animal Experimentation Ethical Committee of the University of Barcelona (CEEA 82/16). A single intravenous (i.v.) dose of the compound (1 mg/kg) or DMSO (control group) was administered. The animals were monitored during 14 days after the inoculation of the treatment. Signs of toxicity such as weight reduction, bristly coat, reduced mobility, and ocular epiphora were verified. The animals were weighed each day until the end of the experiment. A decrease of 80% of the initial weight of the mouse was considered as an endpoint criterion.

## Results

### Synthesis of Gold(III) Complex **3**

There is increasing evidence that gold(III) complexes interact with thiol groups of cysteine residues via ligand exchange (Pratesi et al., 2019; Thomas and Casini, 2020). Thus, we investigated the feasibility of chloride ligands displacement of complex **2** by a thiolate ligand. Treatment of a solution of **2** in aqueous acetonitrile with 1 equiv of D-penicillamine (pa) afforded complex **3** [Au(dppta)(pa)]⋅HCl (Figure 1B). Compound **3** was isolated as a mixture of two diastereomers in a ratio of ca 50/50 due to the bonding of the P-racemic phosphinothioic amide scaffold with the chiral amino acid. Accordingly, duplicated signals were observed in the ^1^H, ^13^C, and ^31^P NMR spectra (Supplementary Figures 4–7). The positive-ion ESI-MS spectrum displayed an intense parent ion [M + H]^+^ with m/z 661.1387 (calculated 661.1381), in agreement with the displacement of both chlorine atoms of **2** by the amino acid. The presence of the amino acid moiety of **3** was ascertained in the ^13^C NMR spectrum through the signals at δ 170.8/170.9 and 29.1/29.2, 29.3/29.9 ppm corresponding to the carbonyl and methyl carbons, respectively. Complex **3** gave crystals of suitable quality for an x-ray structure determination on recrystallization from acetonitrile, and the analysis was carried out in order to unequivocally determine which isomer had been formed. An oak ridge thermal ellipsoid plot (ORTEP) diagram of **3** is shown in Figure 1C (see also Supplementary Figure 8). The asymmetric unit contains two solvent molecules. Selected crystal data are given in Supplementary Table 2. The structure of **3** shows that the gold is square planar, coordinated to the phenylphosphinothioic amide (dppta) moiety forming a five-membered S^∧^C chelate ring, and the 2-aminothiol moiety forming a five-membered S^∧^N chelate ring. The 2-aminothiol ligand is coordinated with the thiolate sulfur *trans* to the S atom of the cycloaurated dppta ligand. To the best of our knowledge, there is no precedent in the literature for gold(III) complex stabilized by bidentate ligands showing this arrangement of sulfur atoms. A cyclometallated-penicillamine gold(III) complex [Au(ppy)(pa)] (ppy = phenylpyridine) has been reported in the literature (Kuwamura et al., 2014).

### Preliminary Antimicrobial Activity Assessment of Gold(III) Complexes **1–3** and Stability of Complex **2**

*In vitro* antimicrobial activity of gold(III) complexes **1** (Oña-Burgos et al., 2009), **2** (Belmonte-Sánchez et al., 2017), and **3** was screened using a limited panel of bacteria representing microorganisms of clinical importance (Table 1). Such bacterial strains have been selected among the most representative Gram-positive and Gram-negative bacteria causing important clinical infections, considering their resistance profile and their ability to form biofilm.

**TABLE 1:** Minimal inhibitory concentration (MIC) values of complexes *1–3* compared with commercial antibiotics.

		MIC (mg/L)				
Strains	Species	1	2	3	CIP	TOB
E3	*Escherichia coli*	>512	32	>512	0.25	8
166097-953	*Pseudomonas aeruginosa*	>512	32	>512	128	128
162065-705	MRSA	>512	4	>512	128	1
FG05015	*Staphylococcus epidermidis*	128	4	>512	16	0.25
8838	*Haemophilus influenzae*	128	32	>512	0.25	1

*MIC, minimal inhibitory concentration; CIP, ciprofloxacin; TOB, tobramycin.*

The minimal inhibitory concentration (MIC) values obtained for the gold(III) complex **1** ranged from 128 to >512 mg/L. Compared with control antibiotics CIP and TOB, with cut off values of resistance of 4 and 16 mg/L, respectively, these pathogens are fully resistant toward **1**. Complex **3** also had no activity against any of the strains (MICs of > 512 mg/L). Interestingly, complex **2** showed MICs ranging from 4 to 32 mg/L for the same set of microorganisms. This means that a minor structural change of a P = O group of **1** by a P = S linkage in **2** produced a significant improvement in the antimicrobial activity. This feature might be associated with the higher aurophilicity of the sulfur atom with respect to the oxygen atom and its contribution to the stabilization of the complex. The remarkable different behavior of complexes **2** and **3** reveals the key role of Au–Cl bonds in the antibacterial activity observed. Subsequent studies were focused on complex **2**. As maintaining the integrity of the metallocenter is of prime importance to correlate any observed biocidal activity with the structure of the complex, the stability of compound **2** under the conditions used for the biological evaluation was assessed. ^31^P NMR monitoring of complex **2** (12 mM) in DMSO-*d*_6_ or CD_3_CN and PBS buffer at 37°C for 18 h showed no signs of gold complex degradation (Supplementary Figure 1 and Supplementary Table 1). However, samples incubated in ISO-Sensitest medium containing small amounts of L-cysteine hydrochloride showed a slight decomposition after 18 h of preparation (Figure 1D and Supplementary Figure 2, new signals of very low intensity at δ_*P*_ 73.0 and 77.3 ppm). To further explore the stability of [Au(dppta)Cl_2_] **2**, solutions in DMSO were treated under mild acidic aqueous conditions (0.1 N HCl). The ^31^P NMR spectra showed that the complex was perfectly stable at those conditions. Remarkably, complex **2** was stable in 5 N methanolic HCl even on heating at 60°C for prolonged times (Supplementary Figure 1). Moreover, complex **2** crystallized from the acidic methanol solution and the x-ray data demonstrated that the gold metallacycle maintained its structural integrity (Belmonte-Sánchez et al., 2017). However, in the presence of 0.1 N NaOH, signs of degradation began to be observed after 3 h of sample preparation and were about 75% when the sample was left standing at room temperature for 24 h (Supplementary Figure 1).

Reduction of gold(III) ions from HAuCl_4_ by amines is a known method of preparing gold(0) nanoparticles (Newman and Blanchard, 2006). Glycine and lysine from peptidoglycan (among other membrane components), antibiotic drugs, and efflux pumps investigated as efflux systems contain amine groups, which could promote the reduction of complex **2**. Hence, we evaluated the stability of complex **2** in the presence of glycine (Figure 1D), CST, and CIP (Supplementary Figure 2 and Supplementary Table 1) as representative compounds containing amine moieties. In all cases, the ^31^P-NMR spectra of samples of **2** containing culture medium and the antibiotics showed that the signal of the gold(III) complex was unaffected after 18 h.

### Antimicrobial Activity of Gold(III) Complex **2**

**FIGURE 2:**
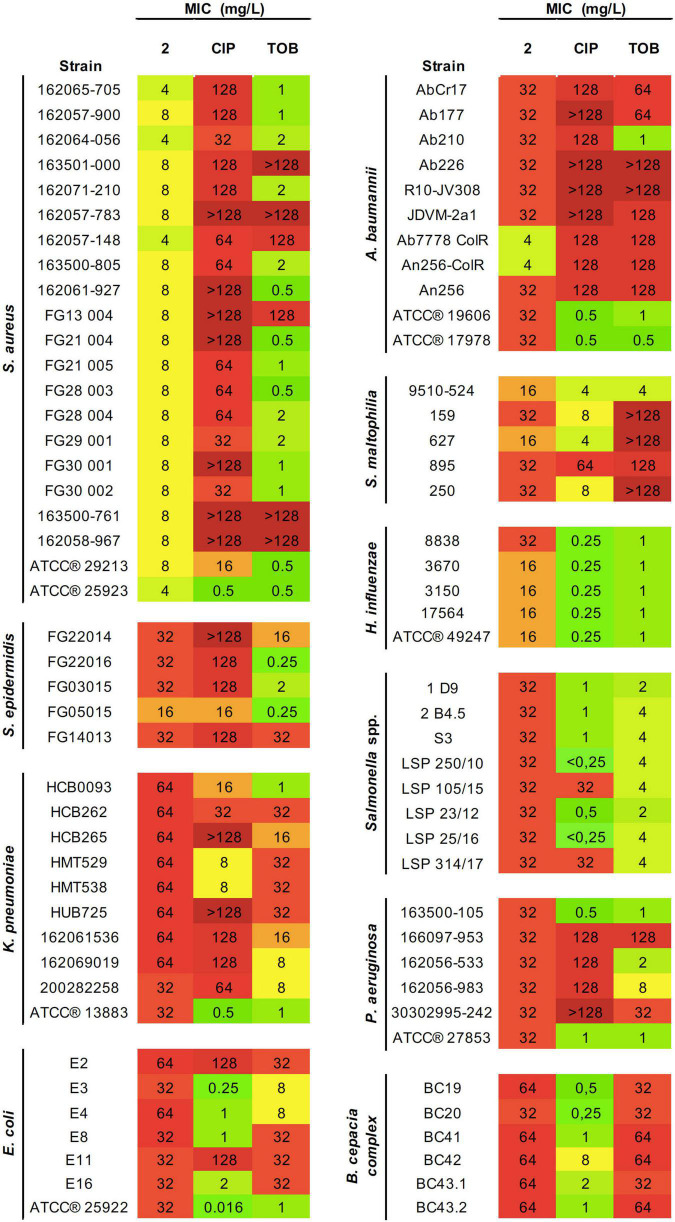
Antibacterial activity of **2** and reference antibiotics against an extended panel of both Gram-positive and Gram-negative pathogens. Minimal inhibitory concentration (MIC) values (mg/L) obtained from three technical and biological replicates are displayed. Color coding indicates concentration ranges: green for lower MICs, orange for medium values, and red for high concentrations. CIP, ciprofloxacin; TOB, tobramycin.

Considering the promising antibacterial activity shown by complex **2**, its effectivity was assessed by MIC testing against a wider range of Gram-positive and Gram-negative pathogens from diverse etiologies, resistance profiles, and biofilm formation abilities (Figure 2). The highest selective activity displayed by **2** was against Gram-positive pathogens, especially against *S. aureus* strains (both MRSA and MSSA) with MIC values ranging from 4 to 8 mg/L. The MIC values against *S. epidermidis* were one- to two-fold higher. The inhibitory activity of **2** against *S. aureus* and *S. epidermidis* strains even approaches or improves the results obtained for therapeutic antibiotics CIP and TOB.

Most of Gram-negative pathogens showed moderate MIC values, in the range of 16–32 mg/L. This includes *S. maltophilia*, *P. aeruginosa*, *H. influenzae, A. baumannii*, *Salmonella* spp., and certain *E. coli*, *K. pneumoniae*, and *B. cepacia* strains. The highest activity was observed for *H. influenzae* strains. However, the greatest effectiveness compared with the antibiotics of reference was found for *A. baumannii*. For most strains tested, complex **2** showed two- to four-fold higher inhibitory efficacy than CIP or TOB. Importantly, this improved activity applies to the pan-resistant *A. baumannii* AbCr17 strain, which shows resistance even to last resort antibiotics such as colistin.

Overall, the ranges of MICs presented by **2** among the different bacterial species were very homogenous. We observed that within a single species, MIC values for the same molecule tend to remain constant, with variations of ±one-fold. Not only gold(III) complexes showed constant MICs among all isolates, but also that concentrations remained constant even against strains with variable resistance profiles, e.g., CIP-resistant *Salmonella* spp. 1 or CIP and ampicillin (AMP)-resistant *Salmonella* spp. LSP 314/17 compared with other *Salmonella* spp. isolates with more sensitive profiles. Both clinical and ATCC^®^ strains exhibited similar susceptibility against **2**.

### Biofilm Inhibitory Activity

**FIGURE 3:**
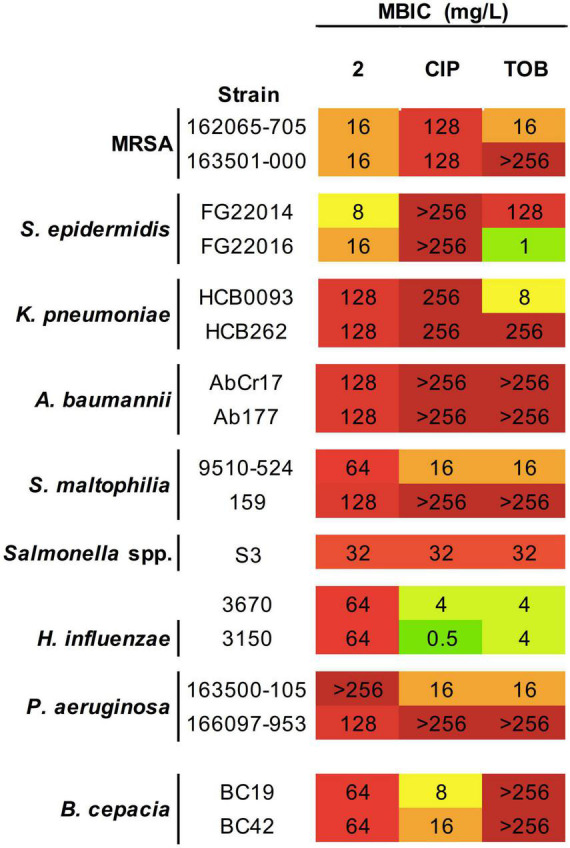
Antibacterial activity of **2** and reference antibiotics against biofilm-forming strains. Minimum biofilm inhibitory concentration (MBIC) values (mg/L) obtained from three technical and biological replicates are displayed. Color coding indicates concentration ranges: green for lower MICs, orange for medium values, and red for high concentrations. CIP, ciprofloxacin; TOB, tobramycin.

Biofilm inhibition formation assays showed MBIC values for **2** of 16–32 mg/L for MRSA, only two-fold higher than the MIC in planktonic phase (Figure 3). In the case of MRSA isolates, MBIC values of **2** are significantly lower than those obtained for CIP and TOB. Interestingly, complex **2** was able to inhibit *S. epidermidis* biofilm formation at concentrations lower than the MIC, suggesting a potent effect on avoiding attachment of adhesion cells in the first phases of biofilm formation.

In Gram-negative strains, MBIC values in *Salmonella* spp. and *B. cepacia* did not vary from their MICs and were the same found for CIP and TOB inhibition of *Salmonella* spp. biofilms or even improve the efficacy more than four times that observed for TOB in *B. cepacia*. On the other hand, *K. pneumoniae* and *A. baumannii* showing MBICs values two and four times greater, respectively, than their corresponding MICs. Remarkably, complex **2** exhibited a higher biofilm inhibitory activity than CIP and TOB, including in the *K. pneumoniae* HCB262 strain resistant to CIP, and the pan-resistant *A. baumannii* AbCr117 strain. For *S. maltophilia* and *P. aeruginosa* strains, the MBICs obtained after exposition to **2** were ≥four-fold higher than their corresponding MIC and showed a strain-dependent biofilm inhibitory activity with only a modest improvement with respect to the reference antibiotics in the best cases.

### Quantification of Gold(III) Complex **2** Bacterial Uptake

The antibacterial activity of a drug is determined by the concentration reaching the target. So, an ICP-MS quantification of the accumulation of complex **2** in MRSA 163501-000 and in *A. baumannii* Cr17 was performed. The time dependent uptake of complex **2** was determined by analyzing samples of the bacteria incubated with **2** at 37°C of 0, 5, and 8 min to minimize bacterial lysis and avoid saturation of the accumulation pathway (Prochnow et al., 2019). Gold determination from the stock solution of complex **2** and PBS buffer were used as controls (Table 2). For *S. aureus*, samples processed right at the time of starting the treatment (T0) showed an average Au content of 0.20 fg/CFU, and the amount of gold accumulated underwent a small increase to 0.26 and 0.27 fg/CFU after 5 and 8 min of incubation with **2**, respectively. The average Au content on the *A. baumannii* lysates was significantly lower: 0.02 fg/CFU was detected at the beginning of the treatment with a slight increase of 0.03 fg/CFU in further 5 and 8 min timepoints.

**TABLE 2:** Quantification of **2** uptake by inductively coupled plasma–mass-spectrometry (ICP-MS) Au detection in bacterial lysates.

	$\bar{x}$Au (μg/ml)	2 (μg/ml)	Total CFU	Au/CFU (fg)	2/CFU (fg)
**MRSA 163501-000**					
C(+) AuC10	6.53 ± 0.28	30	–	–	–
T0	3.57 ± 0.07	16.41	8.90E + 10	0.20	0.92
T5	4.33 ± 0.07	19.76	8.40E + 10	0.26	1.18
T8	4.53 ± 0.15	20.81	8.55E + 10	0.27	1.22
C(−) PBS	0.006 ± 0.01	0.05	8.90E + 10	0.0005	0.002
***Acinetobacter baumannii* AbCr17**					
C(+) AuC10	4.40 ± 0.38	20	–	–	–
T0	0.11 ± 0.03	0.5	2.23E + 10	0.02	0.11
T5	0.15 ± 0.02	0.68	2.20E + 10	0.03	0.15
T8	0.145 ± 0.02	0.65	2.16E + 10	0.03	0.15
C(−) PBS	0.002 ± 0.001	0.009	2.23E + 10	0.0004	0.002

*T, time of sampling in minutes.*

### Analysis of Lyophilized Samples

In order to gain insight about the gold complexes present after treatment of bacteria with complex **2**, a ^31^P-NMR study was undertaken. A long accumulation ^31^P-NMR spectrum (20k scans) of a lyophilizate resulting from the incubation of *A. baumannii* with complex **2** (8 × MIC) showed only the signal of **2** (Supplementary Figure 3). This result indicates that at least the major amount of complex **2** that did not interact with the bacteria remained unchanged.

### Synergy Testing

The synergistic effect of **2** with two conventional antibiotics against representative bacterial strains was investigated using a checkerboard array method, and the effects were evaluated by determination of the FICI. Table 3 shows the checkerboard array results from combining **2** with CST and CIP. In combination with CST, which acts in the cell membrane, complex **2** showed a synergistic effect against all the tested strains, with FICI values below 0.5, including CST-resistant *A. baumannii* AbCr17 and intrinsically CST-resistant MRSA 163501-000. Combined with colistin, the concentration of **2** necessary to inhibit growth is four-fold lower than in monotherapy for *P. aeruginosa*, *A. baumannii*, and *E. coli* strains. In the same way, the inhibitory concentration of colistin is also reduced from two- to four-fold when combined with **2**. In combination with ciprofloxacin, a DNA gyrase inhibitor, **2** did not enhance or disturb its inhibitory activity, resulting in FICI values of 2, considered as indifference. Antagonism was not noted in any of the combinations tested.

**TABLE 3:** Antibacterial activity of **2** in combination with commercial antibiotics.

			MIC (mg/L)				
Bacterial species	Strain	AB	2	AB	2-AB*	FICI^#^	Effect
MRSA	163501-000	CST	8	512	2–64	0.25	Synergy
		CIP	8	128	8–128	2	Indifference
*Pseudomonas aeruginosa*	166097-953	CST	32	1	2–0.25	0.3	Synergy
		CIP	32	128	32–128	2	Indifference
*Stenotrophomonas maltophilia*	895	CST	32	1	4–0.125	0.25	Synergy
		CIP	32	64	32–64	2	Indifference
*Haemophilus influenzae*	3150	CST	32	2	8–0.5	0.5	Synergy
		CIP	32	0.25	32–0.25	2	Indifference
*Acinetobacter baumannii*	AbCr17	CST	32	32	2–2	0.125	Synergy
		CIP	32	128	32–128	2	Indifference
*Escherichia coli*	EC11	CST	32	0.25	2–0.06	0.3	Synergy
		CIP	32	128	32–128	2	Indifference

*MIC, minimal inhibitory concentration; CST, colistin; CIP, ciprofloxacin.*

*^#^FICI = (MIC_drug A_ in combination/MIC_drug A_ alone) + (MIC_drug B_ in combination/MIC_drug B_ alone).*

*Lowest concentrations of both drugs at which the combination had an inhibitory effect.

### Time-Dependent Killing Assays

**FIGURE 4:**
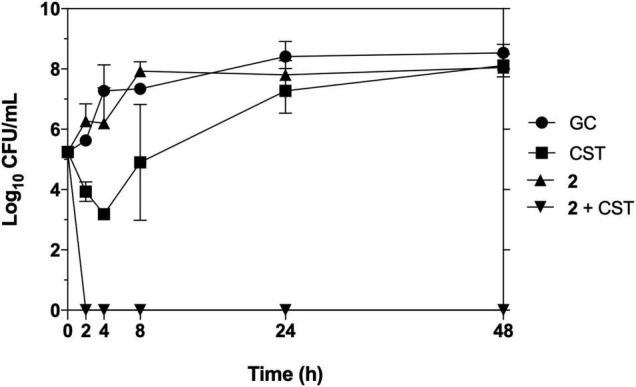
Time-kill kinetics of **2** in combination with colistin against pan-resistant strain *Acinetobacter baumannii* Cr17. Graph shows the lowest bactericidal combination: colistin (CST) at 0.03 × MIC (1 mg/L), **2** at 0.125 × MIC (4 mg/L).

To corroborate the synergistic results of **2** with colistin on the checkerboard arrays, a time-kill kinetic assay against pan-resistant *A. baumannii* AbCr17 in a macrodilution series was next performed. Complex **2** and colistin were both combined in a wide range of concentrations (from 0.03 × MIC to 2 × MIC). Colistin and **2** at 1 and 4 mg/L, respectively, were the lowest concentrations at which synergistic bactericidal effect was observed. This clearance effect appears in less than 2 h when the test population was reduced below the limit of detection, with no signs of rebound growth after 48 h. Both colistin and **2** in monotherapy at the same concentrations did not achieve a ≥2 log bacterial reduction from the initial inoculum at any time point, following a similar trend as the untreated control (Figure 4). It is to note that this synergistic effect between **2** and colistin allows reducing therapeutic concentrations of both drugs.

### Gyrase and Topoisomerase IV Inhibition

It has been previously described that some N^∧^N^∧^N^∧^N-Au(III) complexes (Gupta et al., 2018) and C^∧^N^∧^C-Au(IIII) cycloaurates (Castelli et al., 2011) exert their activity by inhibiting human replication topoisomerases I and II. In bacteria, gyrase and topoisomerase IV are the main enzymes involved in DNA replication, which are also common targets for several commercial antimicrobials, such as fluoroquinolones. DNA gyrase is a type II topoisomerase that induces negative DNA supercoiling and is highly conserved in all bacteria but absent in eukaryotes. However, topoisomerase IV is responsible of decatenating interlinked double-stranded DNA following replication.

**FIGURE 5:**
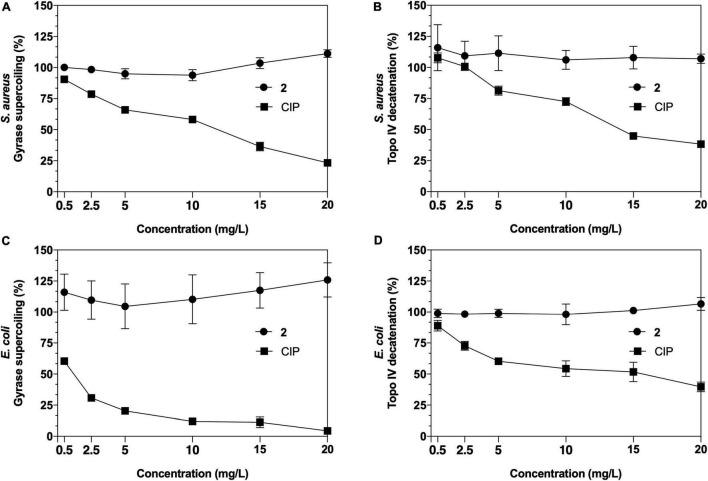
Results of *Staphylococcus aureus* and *Escherichia coli* gyrase supercoiling **(A,C)** and topoisomerase IV decatenation **(B,D)** inhibition assays of **2** and ciprofloxacin (CIP) as a control.

To determine whether the antimicrobial activity of **2** had a direct effect in inhibiting these replication enzymes in both Gram-positive and Gram-negative pathogens, a series of *in vitro* quantitative gel-based assays against purified *S. aureus* and *E. coli* gyrase and topoisomerase IV enzymes were performed (Figure 5). For gyrase supercoiling inhibition assays (Figures 5A,C), no alteration in the enzyme activity was observed when **2** was added in an increasing range of concentrations in both *S. aureus* and *E. coli*, maintaining its activity over 100% throughout the experiment when compared with the untreated enzyme control. Similarly, no reduced activity was noted in topoisomerase IV decatenation assays (Figures 5B,D) at any of the concentrations against *S. aureus* or *E. coli* topoisomerases IV. These enzyme inhibition assays demonstrate that neither DNA gyrase nor topoisomerase IV are bacterial targets of the antimicrobial activities of **2**.

### Activity of Gold(III) Complex **2** in the Presence of Efflux Pump Inhibitors

Drug efflux pumps are commonly expressed in Gram-positive and Gram-negative pathogens, playing a major role in antimicrobial resistance extruding antimicrobials outside the bacterial cell (Ughachukwu and Unekwe, 2012). It is therefore significant to determine the effect of efflux systems on the activity of new antimicrobials. In this study, we evaluated the effect over complex **2** antimicrobial activity of three major EPIs: PaβN, a broad-spectrum efflux inhibitor (especially of Gram-negative bacteria) due to its behavior as a cationic peptide (Ughachukwu and Unekwe, 2012); CCCP, which blocks proton-dependent electrochemical gradients (Park and Ko, 2015); and RSP, which inhibits mostly efflux systems of the resistance–nodulation-cell division family (Garvey and Piddock, 2008). According to our results, the presence of subinhibitory concentrations of these three EPIs did not increase the activity of **2** in any of the *S. aureus*, *S. maltophilia*, *P. aeruginosa*, *H. influenzae*, and *E. coli* strains tested, for which the original MIC values remained unaltered. Remarkably, when CCCP was added to **2**, a complete loss of activity was observed (MIC values > 128 mg/L) only against Gram-negative bacteria (except *H. influenzae*).

### Transmission Electron Microscopy Analysis

Transmission electron microscopy analysis revealed significant ultrastructural differences in *S. aureus*, *A. baumannii*, and *P. aeruginosa* morphology upon exposure to **2** at MIC concentrations compared with the untreated controls. However, the type of damage observed differed between species.

**FIGURE 6:**
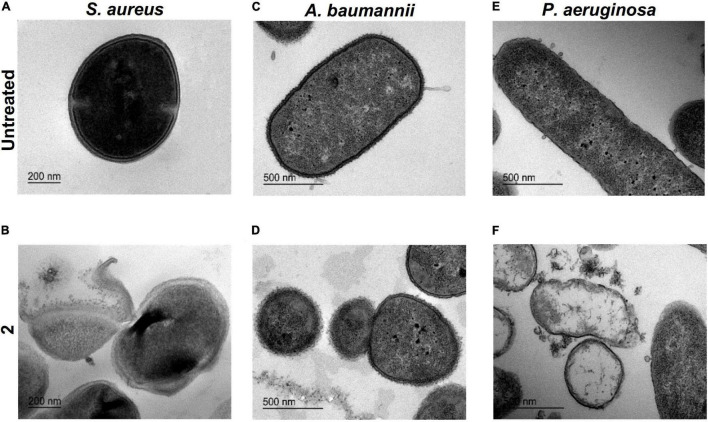
Transmission electron microscopy imaging. Ultrastructural analysis of *Staphylococcus aureus* untreated **(A)** and exposed to **2 (B)** (bar = 200 nm). *Acinetobacter baumannii* untreated **(C)** and exposed to **2 (D)** (bar = 500 nm). *Pseudomonas aeruginosa* untreated **(E)** exposed to **2 (F)** (bar = 500 nm). In all cases, a concentration of **2** equal to the minimal inhibitory concentration (MIC) was used.

In the untreated control images (Figure 6A), *S. aureus* appeared with its characteristic cocci shape, well-differentiated membranes, and condensed cytoplasm. When treated with **2** (Figure 6B), *S. aureus* showed an emphasized membrane rupture, exhibiting some roughness on the wall that might indicate membrane destabilization with subsequent cell deformation. We also observed a marked cytoplasmic retraction, membrane disintegration, and intracytoplasmic condensation compared with the untreated control. In *A. baumannii* cells exposed to **2** (Figure 6D), we noticed an obvious membrane disintegration, a clear diffusion of the outer and inner membranes compared with the unbroken well-differentiated cell walls observed in the untreated control (Figure 6C). In both images, the cytoplasm appears almost intact. *P. aeruginosa* showed a slightly different behavior compared with *A. baumannii* when exposed to treatment with **2**. Compared with the untreated control (Figure 6E), *P. aeruginosa* exposed to **2** showed evident cell damage and accumulation of ruptured cell debris. In treated cells (Figure 6F), we noticed a complete leakage of the cytoplasmic content and partial degradation of the cell wall, proceeding into empty cells and altering the classical *Pseudomonas* rod shape.

### Bacterial Membrane Permeabilization Assays

**FIGURE 7:**
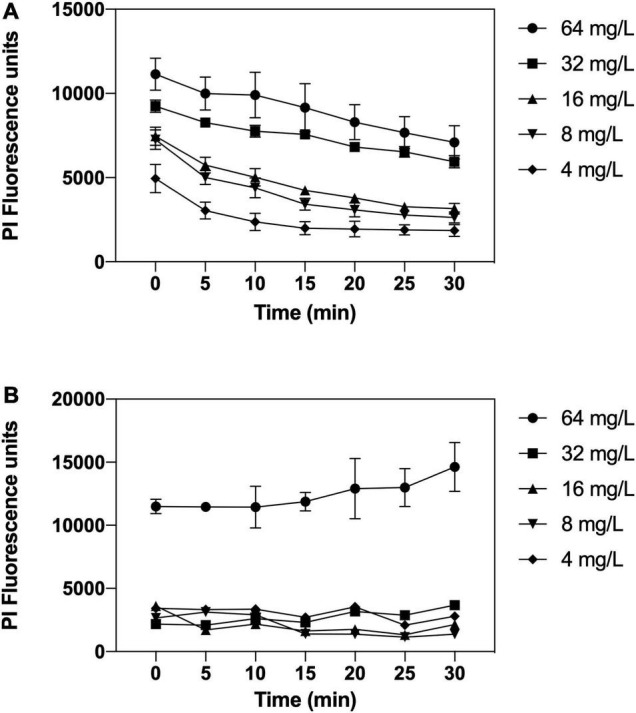
Membrane permeabilizations assays by complex **2** against MRSA 163501-000 **(A)** and *Acinetobacter baumannii* Cr17 **(B)**. Bacterial cells were labeled with SYTO9 and propidium iodide (PI) dyes; treated with complex **2** (4–64 mg/L), and the PI fluorescence was measured at 5-min intervals posttreatment. Data represent the mean ± SD of the PI fluorescence signal after subtracting the untreated control PI signal.

To further explore the interaction of complex **2** with the bacterial membrane, permeabilization assays were carried out using the LIVE/DEAD viability kit, containing SYTO9 and PI dyes. SYTO9 is permeable to the membrane and labels intact and damaged cells, while only membrane-compromised bacteria are labeled by the impermeable PI, reducing the SYTO9 fluorescence. On the membrane permeabilization assay in MRSA 163501-000 (Figure 7A), our data show that complex **2** is effective in permeabilizing the membrane at all the concentrations tested. We observed an increased PI fluorescence signal within the entire 30-min interval after treatment with **2**, which also increased at higher concentrations of the compound. Complex **2** is less effective at permeabilizing the *A. baumannii* membrane (Figure 7B); we only detected a significative increase in the PI fluorescence signal at 64 mg/L.

### Point of Resistance

**FIGURE 8:**
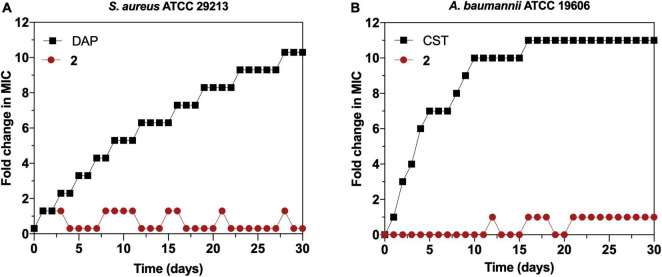
**(A)** Resistance development of *Staphylococcus aureus* ATCC 29213 during serial passaging in the presence of sub-minimal inhibitory concentration (MIC) levels of **2** with daptomycin (DAP) as a control. **(B)** Resistance development of *Acinetobacter baumannii* ATCC 19606 during serial passaging in the presence of sub-MIC levels of **2** with colistin (CST) as a control. For colistin, 2,048 × MIC was the highest concentration tested. Graphs are representative of two independent experiments. The y-axis represents the fold increase in the MIC value after each daily serial passage compared with the parental strain.

We continued exploring the antimicrobial activity of **2** favoring the selection of resistant mutants by sequential step mutation in both Gram-positive (*S. aureus* ATCC 29213) (Figure 8A) and Gram-negative (*A. baumannii* ATCC 19606) (Figure 8B) bacteria. After 30 daily sequential passages and daily checking for >one-fold changes in the MIC of **2**, no resistant mutants were obtained at any timepoint. In contrast, daily checking for >one-fold changes in MIC detected resistant mutants for DAP (in *S. aureus*) and colistin (CST) (in *A. baumannii*) at day 3 of sequential culturing, and reached MICs 10-fold higher than the parental strain by the end of the experiment. Therefore, serial passage of *S. aureus* and *A. baumannii* in the presence of sub-MIC levels of **2** over a period of 30 days failed to develop resistant mutants.

### Cytotoxicity

**FIGURE 9:**
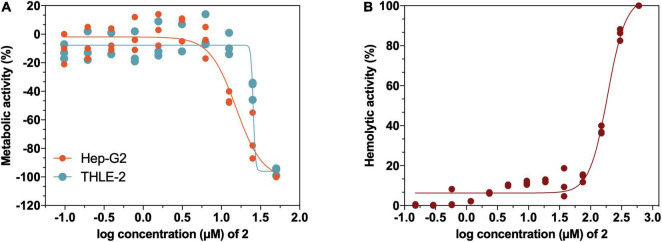
**(A)** Cytotoxicity profile of **2** in liver non-tumoral THLE-2 and tumoral Hep G2 cell lines determined by 3-(4,5-Dimethylthiazol-2-yl)-2,5-diphenyltetrazolium bromide (MTT) assay to detect loss of metabolic activity. **(B)** Hemolytic activity of **2** on human erythrocytes from a healthy donor.

Considering the potential of **2** as a therapeutic antimicrobial agent, the *in vitro* toxicity profile of **2** in tumor- and non-tumor-derived liver cell lines from liver tissue, as well as its hemolytic activity in human erythrocytes was evaluated. The results of the *in vitro* cytotoxicity after a 72-h exposure show an IC_50_ value of 25.5 μM (15 mg/L) in non-tumor THLE-2 cell line, whereas the toxicity profile using the tumor Hep G2 cell line was slightly less favorable with an IC_50_ value of 15.42 μM (9 mg/L) (Figure 9A). Regarding the hemolysis assays (Figure 9B), no release of hemoglobin was detected in the usual experimental concentrations of **2** (<75 μM, 43.8 mg/L), showing an IC_50_ value of 187.8 μM (109.7 mg/L), which indicates that erythrocytes show high tolerance to **2**. Overall, these results point to the limited toxicity of **2** at therapeutic concentrations in mammalian cell lines, confirming its safety for the following *in vivo* studies.

### *In vivo* Acute Toxicity

**FIGURE 10:**
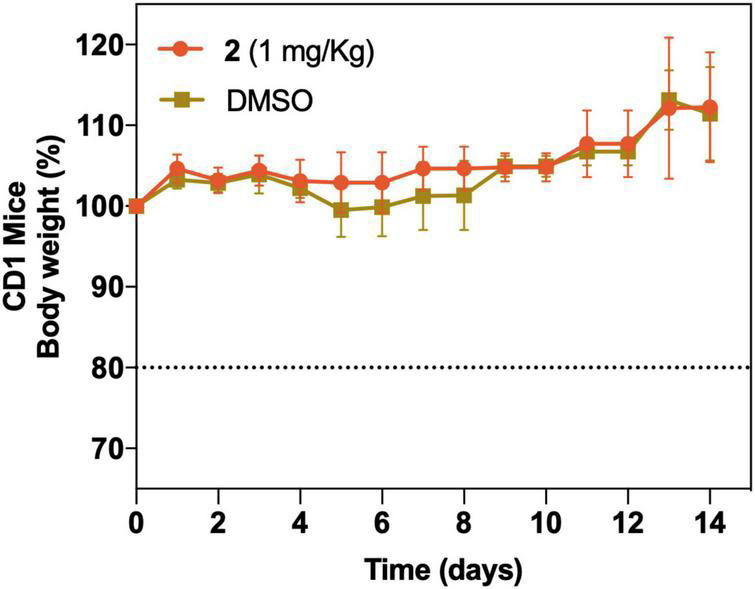
*In vivo* acute toxicity of a single i.v. dose of **2** at 1 mg/kg (*n* = 5) and vehicle control group (DMSO) (*n* = 5). Body weight (as well as other visual toxicity signs) was monitored for 14 days posttreatment. The dotted line represents a 20% body weight loss, which was considered as an endpoint criterion.

As a preliminary assessment of acute toxicity of **2** *in vivo*, CD1 mice were infused with a single i.v. dose of 1 mg/kg. Full survival of the study group with no significant weight loss group (DMSO) or visual signs of toxicity was detected during the course of the experiment compared with the control (Figure 10).

## Discussion

The emergence and spread of antimicrobial resistance in the last decades have led to a situation in which current antimicrobials have lost their effectiveness toward pathogens that have become untreatable with last resort antibiotics and represent a major global health threat. In this scenario, the search for new antimicrobials and treatments is mandatory, but traditional methods employed in drug discovery are time consuming and financially challenging. Repurposing old drugs for new applications seems an adequate strategy for researchers to overcome this situation (Miró-Canturri et al., 2019), with successful examples, such as the gold complex auranofin, which was initially approved as an antirheumatic, but it was not until recently that its potent antibacterial activity has been described especially against Gram-positive pathogens (Thangamani et al., 2016; Hutton et al., 2020). In the same wave, gold(III) complexes extensively studied as antitumorals (Bertrand et al., 2018) are emerging as potential antimicrobial drugs (Pacheco et al., 2009; Glišić and Djuran, 2014). Nevertheless, studies on gold(III) complexes activity toward bacteria are still scarce, considering also the great diversity within these molecules regarding their general structure and ligands (Glišić and Djuran, 2014). To the extent of our knowledge, the present study reports first evidence on S^∧^C cyclometallated gold(III) complex **2** as antimicrobial and biofilm inhibitory agents.

The antimicrobial activity of the gold(III) complexes **1**–**3** was investigated against an array of clinically relevant Gram-positive and Gram-negative strains. Only complex **2** showed antibacterial activity; therefore, further studies were carried out only with this molecule. Even though complex **3** lacks antimicrobial activity, its formation provides strong support to the hypothesis of the mechanism of action of complex **2** involving the binding to thiol groups of proteins containing cysteine amino acids via nucleophilic displacement of chloride. On the one hand, the complex is stable, i.e., once formed, it does not undergo additional transformations such as reductive elimination to Au(I) derivatives. On the other, the lack of activity indicates that the antibacterial activity is due to the inhibition of the function of the protein(s), which underwent the binding to Au(III). The results on the antimicrobial activity of complex **2** against a wider panel of clinical isolates (Figure 3) show that **2** exerts potent antimicrobial activity against Gram-positive strains, especially MRSA and MSSA (MIC ≤ 8), compared with other commercial antibiotics. This certain degree of specificity for *S. aureus* is consistent with previous findings for structurally related C^∧^N-cycloaurated gold(III) complexes [Au(damp)Cl_2_] and [Au(ppy)Cl_2_] (Parish et al., 1996; Parish, 1999), but it was not the case for mononuclear gold(III) complexes [AuCl_3_(N-heterocycle)], which showed moderate activity toward *S. aureus* strains (Savić, 2016). Complex **2** also showed similar or higher antibacterial activity compared with [Au(damp)Cl_2_] and [Au(ppy)Cl_2_] against *E. coli* and *P. aeruginosa*. Overall, the MIC values of **2** against the panel of Gram-negative pathogens assayed are mostly in the medium range of antibacterial activity and are higher than those of the reference antibiotics, with the exception of certain *A. baumannii* strains. These results are especially significant considering that the higher efficacy of **2** compared with CIP and TOB is also observed in the pan-resistant strain AbCr17. This preference for Gram-positive pathogens and stronger activity toward *A. baumannii* has been recently reported for gold NHC complexes and also for the reference gold(I) complex drug auranofin, together with a consistently higher antibacterial activity noted for gold(III) NHC complexes, compared with gold(I) analogs (Büssing et al., 2021).

Regarding the antibiofilm activity, reports show that several gold(III) complexes exert prominent biofilm-disrupting activity against strains with high resistance to antibiotics (Coates et al., 2011). As an example, gold(III) complexes with *N*-donor ligands have antibiofilm activity, causing a reduction above 50% of biofilm when the compound was applied before the adhesion phase of the *P. aeruginosa* strains was analyzed (Radulović et al., 2018). According to our results, **2** affected biofilm formation of Gram-positive strains when added in the preadhesion phase, especially against *S. epidermidis* strains showing lower MBIC values compared with their planktonic activity, probably affecting their attachment to the surface and compromising this trait of the *S. epidermis* pathogenicity. Conversely, **2** rarely affected biofilm formation among the Gram-negative species tested. However, it is important to note that, in the case of *A. baumannii*, the MBIC of **2** is lower than those from CIP and TOB, and *Salmonella* strains showed similar MIC and MBIC values for the complex **2**, being the latter similar to the MBICs obtained with CIP and TOB. The comparatively lesser effect of **2** observed both in planktonic and in biofilm growth on Gram-negative bacteria seems to be a common trait for gold(III) complexes or even gold-based drugs (Parish et al., 1996; Parish, 1999; Hutton et al., 2020), with very few exceptions that have pointed some selectivity only toward *P. aeruginosa* strains (Goss et al., 2003; Radulović et al., 2018). A previous work using auranofin plus polyurethane-coated catheters, showed that this material was able to inhibit completely the MRSA *in vitro* biofilm formation. However, this effect was not observed with auranofin and polyurethane alone (Liu et al., 2019).

Therefore, even if the MIC values obtained are comparable or even lower than commercial antibiotics for MDR strains, it appears that the outer membrane is compromising the activity of **2**. The detection of significant amounts of gold a few seconds after mixing the bacterial cell culture with the solution of **2** suggests that a binding of the gold cation to bacterial membrane components through a fast reaction has taken place, most probably via Au–S bond-forming reactions with cysteine residues of bacterial cell envelope proteins (Venezia et al., 2005; Desvaux et al., 2006). The synthesis of complex **3** upon the reaction of gold(III) dichloride **2** with D-penicillamine substantiate the feasibility of these in-cell transformations. Although there was an evident Au uptake compared with the untreated controls, **2** accumulated to a lower extent than other standard antibiotics in similar studies (Prochnow et al., 2019), and the small increase in gold uptake within time supports a fast saturation of the accumulation pathway.

The fact that the outer membrane serves as a barrier toward **2** was also corroborated by the synergistic effect observed when **2** was combined with colistin in all Gram-negative strains tested. When colistin permeabilizes the bacterial outer membrane (Mohamed et al., 2016), the activity of **2** is greatly enhanced, as checkerboard and time-kill kinetics assays have demonstrated, even achieving a bactericidal effect against the pan-resistant *A. baumannii* AbCr17. The combination managed to reduce at least three times the effective concentration of **2** compared with its MIC value; the amount of colistin administered in combination to treat a colistin-resistant strain is reduced from 32 to 1 mg/L, which is below the CLSI breakpoint for colistin against *A. baumannii* (CLSI, 2020). Overall, this significant treatment not only sheds light on the mechanism of action of **2** but also could compensate for the lack of direct antimicrobial activity of **2** against Gram-negative bacteria. Moreover, the remarkable reduction of colistin therapeutic dose when administered in combination with **2** diminish notably the risk associated with the hepatotoxicity and nephrotoxicity of the antibiotic. No previous data on Au(III) complexes combined with colistin were found in the literature. Recently, it has been reported that auranofin acted synergistically with colistin against a wide spectrum of carbapenem- and/or colistin-resistant bacterial strains (Sun et al., 2020). Synergistic studies between colistin and other transition-metal complexes have been described only for Mn(I) (Betts et al., 2017) and Ga(III) (Choi et al., 2019). Very few studies evaluate the activity of gold(III) complexes with other commercial antibiotics, one of them reporting the antimicrobial effectivity of an Au(III)–norfloxacin complex [Au(NOR)_2_(H_2_O)_2_]Cl_3_, with moderate activity against *P. aeruginosa* and inactive against *E. coli* (Refat, 2007). In our study, no positive or negative interaction was noted when **2** was combined with ciprofloxacin. These results are in line with our observations in the topoisomerase IV and gyrase inhibition assays, where the presence of **2** did not alter the function of these replication enzymes.

The confirmation of the important role that cell membranes play in the activity of **2** led us to determine the potential effect of drug efflux systems in the mechanism of action of this gold(III) complex. We observed that the activity of complex **2** was not enhanced when coadministered with three major EPIs, which target efflux systems that confer resistance, i.e., to tetracyclines (RSP), fluoroquinolones and other β-lactams (PaβN), and colistin (CCCP). Therefore, complex **2** appears not as a substrate for these efflux pump systems, meaning also that preexisting efflux-mediated resistance should not affect its activity. Conversely, the presence of subinhibitory concentrations of CCCP in some Gram-negative strains produced an inactivation of complex **2**. This could be explained likewise to the reaction of **2** with penicillamine, considering that CCCP has amine-type N–H bonds that can reduce Au(III) to Au(I) or displace the Cl of the gold complex through nucleophilic attack of the nitrogen atom, suppressing its antibacterial activity. TEM microscopy allows to visually identify ultrastructural changes in bacteria when exposed to a target compound. In our TEM optical sections, we clearly observed the effect of **2** on the cell membrane, where cells treated with **2** had a bacterial disruption effect in MRSA; a complete loss of membrane integrity with markedly diffuse inner and outer membranes in *A. baumannii* or a complete leakage of cytoplasmic content as observed in *P. aeruginosa*. Further studies on the effect of complex **2** on the bacterial membrane revealed that **2** causes a quick membrane permeabilization in MRSA, which also relates with the immediate bacterial uptake described above. In this assay, we observed a slight reduction of the PI fluorescence over time, probably due to the loss of bacterial morphology and density caused by the treatment itself. The permeabilizing activity of **2** against *A. baumannii* was achieved only at high concentrations, which also correlates with its antibacterial activity. In this case, we did not observe a decrease in the fluorescence signal over time, as the *A. baumannii* cytoplasm remains intact after the treatment with **2**, as TEM images reveal. These TEM and permeabilization results point to a possible interaction of **2** with the cell membrane that causes membrane disintegration, permeabilization, severe bacterial damage, and subsequent cell death. However, the bacterial uptake detected of complex **2** and its synergism with the Trojan horse-like antibiotic CST could suggest also an intracellular effect of **2**. Thus, additional in-cell targets cannot be fully discarded.

To further support its mode of action, we attempted to obtain mutation frequencies of **2** in *S. aureus* and *A. baumannii*. After performing 30 daily sequential passages of both bacteria on subinhibitory concentrations of **2**, no resistant mutants were obtained. Although there are no previous point of resistance studies for gold(III) complexes in the current literature, some studies with similar gold-based drugs report the same inability to obtain resistant mutants (Harbut et al., 2015; Thangamani et al., 2016). This lack of resistance development through mutations has been described for vancomycin, which acts in the peptidoglycan layer, but it is also often related to drugs with multiple bacterial targets, unspecific mode of action, or/and elevated toxicity (Ling et al., 2015). Nevertheless, **2** showed no hemolytic activity and *in vitro* toxicity at therapeutic concentrations in non-tumoral and tumoral THLE-2/HepG2 liver cells. The slightly increased toxicity over HepG2 cells could be related to the antitumoral activity commonly associated with gold(III) complexes (da Silva Maia et al., 2014). Preliminary *in vivo* toxicity results were particularly promising since **2** showed no acute toxicity signs in concentrations far above the therapeutic range. These results are encouraging for further assessing the pK/pD and the activity *in vivo* of **2** in infection models.

To our knowledge, our study is the first to characterize the antimicrobial activity and *in vitro* and *in vivo* toxicity of a C^∧^S-cycloaurated gold(III) complex from a new chemical class. Complex **2** demonstrates potential as an antimicrobial agent, effective against Gram-positive bacteria (including MRSA) in monotherapy and also against MDR Gram-negative pathogens in combination with CST. This combination enhances the activity of both drugs and allows to significantly reduce their therapeutic concentrations, which is particularly interesting in the case of CST to avoid the emergence of resistance and reduce its well-known nephrotoxicity. Based on the novelty of our findings, we suggest a membrane-related mechanism of action of complex **2** through binding directly to the cysteine residues of the bacterial membrane, although further intracellular targets should be studied to fully decipher its mechanism of action. Given its antibacterial and biofilm inhibitory activity, and considering the highly resistant profile of the strains here tested, its inability to produce resistant mutants, and its promising *in vitro* and *in vivo* toxicity, complex **2** can be considered as the first representative of a new family of antibacterial agents. Additional studies of derivatization of complex **2** for improving the antimicrobial performance are guaranteed.

## Data Availability Statement

The original contributions presented in the study are included in the article/Supplementary Material, further inquiries can be directed to the corresponding authors.

## Ethics Statement

The animal study was reviewed and approved by the Animal Experimentation Ethical Committee of the University of Barcelona (CEEA 82/16).

## Author Contributions

SS and FL-O: conceptualization. CR, VC, RS, YN, MV-DA, MI, and FL-O: methodology. CR: data analysis. CR, FL-O, RS, and SS: writing−original draft preparation. CR, FL-O, FL, and SS: writing−review and editing. All authors have read and agreed to the published version of the manuscript.

## Conflict of Interest

The authors declare that the research was conducted in the absence of any commercial or financial relationships that could be construed as a potential conflict of interest.

## Publisher’s Note

All claims expressed in this article are solely those of the authors and do not necessarily represent those of their affiliated organizations, or those of the publisher, the editors and the reviewers. Any product that may be evaluated in this article, or claim that may be made by its manufacturer, is not guaranteed or endorsed by the publisher.
