# Supplementary material for: A C∧S-Cyclometallated Gold(III) Complex as a Novel Antibacterial Candidate Against Drug-Resistant Bacteria
Source: Front Microbiol. 2022 Mar 3;13:815622. doi: 10.3389/fmicb.2022.815622 (PMC8928146; doi:10.3389/fmicb.2022.815622)
Supplement: Supplementary file 1 [file Data_Sheet_1.docx]

**Supporting Information**

**A C˄S-cyclometallated gold(III) complex as a novel antibacterial candidate against drug-resistant bacteria.**

**Carlos Ratia^1^, Virginio Cepas^1^, Raquel Soengas^2^, Yolanda Navarro^2^, María Velasco de Andrés^3^, María José Iglesias^2^, Francisco Lozano^3^, Fernando López-Ortiz^2†*^, Sara M. Soto^1†*^**

^1^ISGlobal, Hospital Clínic—Universitat de Barcelona, 08036 Barcelona, Spain.

^2^Área de Química Orgánica, Centro de Investigación CIAIMBITAL, Universidad de Almería, 04120 Almería, Spain.

^3^August Pi i Sunyer Biomedical Research Institute (IDIBAPS), Barcelona, Spain.

**^†^** These authors have contributed equally to this work and share senior authorship.

**Contents**

**X-ray studies** S2

^1^H NMR of complex **2** in different media S2

[2-((Diisopropylamino)(phenyl)phosphorothioyl)phenyl] [D-Penicillamine] gold(III) **(3)** S3

**Table S1**. ^31^P NMR chemical shifts of [Au(dppta)Cl_2_] **2** under the different conditions used S4

**Figure S1**. Comparison of ^31^P NMR spectra (121.5 MHz) of [Au(dppta)Cl_2_] **2** under the different conditions used S4

**Figure S2**. Comparison of ^31^P NMR spectra (121.5 MHz) of [Au(dppta)Cl_2_] **2** in the presence of glycine, colistin and ciprofloxacin S5

**Figure S3**. ^31^P NMR spectrum (242.95 MHz MHz) in DMSO-*d*_6_ of a lyophilisate resulting from the incubation of *A. baumannii* with complex **2** (8xMIC) during 1 h S6

**Figure S4.** ^1^H NMR spectrum (500.13 MHz, CD_3_CN) of compound **3**. S6

**Figure S5**. ^13^C NMR spectrum (125.76 MHz, CD_3_CN) of compound **3** S7

**Figure S6**. ^31^P NMR spectrum (202.46 MHz, CD_3_CN) of compound **3**. S7

**Figure S7**. ^1^H,^15^N HMQC spectrum (500.13 MHz, CD_3_CN) of compound **3** S8

**Figure S8**. X-ray crystal structure of **3** (thermal ellipsoids shown at 30% probability) including atomic numbering. Hydrogen atoms have been omitted for clarity. S8

**Table S2**. Selected crystal data for compound **3**. S9

**References** S9

**X-ray studies**

Single crystal X-ray diffraction data were collected on a Bruker D8 Venture diffractometer at 100 K, using CuKα (λ = 1.54178 Å) radiation. Data collection and cell refinement were performed with Bruker APEX3 (Bruker 2014), and Bruker Saint (Bruker 2017), respectively. Data reduction was carried out using SAINT. Empirical multiscan absorption correction using equivalent reflections was performed with the SADABS program (Bruker 2016). The structure solutions and full-matrix least-squares refinements based on F2 were performed with SHELXL program package (Sheldrick 2008). All atoms except for hydrogen were refined anisotropically. Hydrogen atoms were treated by a mixture of independent and constrained refinement. CCDC 2094505 contain the supplementary crystallographic data for this paper. These data can be obtained free of charge from The Cambridge Crystallographic Data Centre via http://www.ccdc.cam.ac.uk/products/csd/request/ (or from Cambridge Crystallographic Data Centre, 12 Union Road, Cambridge, CB2 1EZ, UK (fax: + 44- 1223-336-033; E-mail: deposit@ccdc.cam.ac. uk). The structures were drawn by ORTEP-3 (Farrugia 2012).

**^1^H NMR of complex** **2** **in different media**

**[2-((Diisopropylamino)(phenyl)phosphorothioyl)phenyl] gold(III) dichloride (2).** Complex **2** has been synthesized following a procedure previously reported (Belmonte-Sánchez et al, 2017).

^1^H NMR (DMSO-*d*_6_, 300.13 MHz): δ 1.18 (d, 6H, ^3^*J*_HH_ 6.9 Hz, H12), 1.23 (d, 6H, ^3^*J*_HH_, 6.9 Hz, H13), 3.70 (dsep, 2H, ^3^*J*_PH_ 18.8, ^3^*J*_HH_ 6.9 Hz, H11), 7.52 (m, 2H, H5, H4), 7.72 (m, 1H, H6), 7.76 (td, 2H, ^3^*J*_HH_ 7.8, ^4^*J*_PH_ 4.1 Hz, H9), 7.85 (td, 1H, ^3^*J*_HH_ 7.4, ^4^*J*_PH_ 2.1 Hz, H10), 8.17 (m, 1H, H3), 8.21 (dd, 2H, ^3^*J*_PH_ 14.7, ^3^*J*_HH_ 7.5 Hz, H8) ppm.

^1^H NMR (DMSO-*d*_6_/aq. HCl 0.1 M, 300.13 MHz): δ 1.13 (d, 6H, ^3^*J*_HH_ 6.7 Hz, H12), 1.17 (d, 6H, ^3^*J*_HH_, 6.7 Hz, H13), 3.67 (dsep, 2H, ^3^*J*_PH_ 17.8, ^3^*J*_HH_ 6.8 Hz, H11), 7.48 (m, 2H, H5, H4), 7.61 (m, 1H, H6), 7.70 (m, 2H, H9), 7.80 (td, 1H, ^3^*J*_HH_ 7.4, ^4^*J*_PH_ 2.1 Hz, H10), 8.07 (m, 1H, H3), 8.13 (ddd, 2H, ^3^*J*_PH_ 14.6, ^3^*J*_HH_ 7.5 Hz, H8) ppm.

^1^H NMR (5 M HCl in MeOD, 500 MHz): δ 1.20 (d, 6H, ^3^*J*_HH_ 6.8 Hz, H12), 1.23 (d, 6H, ^3^*J*_HH_, 6.8 Hz, H13), 3.71 (dsep, 2H, ^3^*J*_PH_ 18.3, ^3^*J*_HH_ 6.8 Hz, H11), 7.44 (m, 2H, H4, H5), 7.67 (m, 3H, H9, H10), 7.77 (m, 1H, H10), 8.18 (m, 3H, H3, H8) ppm.

**[2-((Diisopropylamino)(phenyl)phosphorothioyl)phenyl] [D-Penicillamine] gold(III) (3).** To a solution of Au(III) dichloride complex **2** (114 mg, 0.20 mmol) in acetonitrile (8 mL), a solution of D-penicillamine (29 mg, 0.20 mmol) in water (1.2 mL) was added. The reaction mixture was stirred for 1 h and then evaporated. The residue was washed with water and diethyl ether to afford desired complex **3** (122 mg, 85%). Mp: 224-226 ºC. IR (KBr, ν cm**^−^**^1^): 581 (P**=**S), 1439 (CO_2_^-^), 1628 (CO_2_^-^), 1722 (CO_2_H), 2972 (CO_2_H), 3490 (NH_2_). ^1^H NMR (500.13 MHz, CD_3_CN) δ 1.14 (d, 6H, ^3^*J*_HH_ 7.2 Hz, H12), 1.15 (d, 6H, ^3^*J*_HH_ 7.2 Hz, H13), 1.17 (d, 6H, ^3^*J*_HH_ 7.2 Hz, H12’), 1.19 (d, 6H, ^3^*J*_HH_ 7.2 Hz, H13’), 1.28 (s, 3H, H17), 1.53 (s, 3H, H17’), 1.59 (s, 3H, H18’), 1.61 (s, 3H, H18), 3.62 (m, 2H, H11/H11’), 3.68 (m, 2H, H11/H11’), 4.01 (m, 1H, H15), 4.04 (m, 1H, H15’), 5.15 (t, 1H, ^3^*J*_HH_ 10.7 Hz, H19), 5.32 (t, 1H, ^3^*J*_HH_ 9.5 Hz, H19’), 7.33 (t, 1H, ^3^*J*_HH_ 7.7 Hz, H4), 7.34 (t, 1H, ^3^*J*_HH_ 7.7 Hz, H4’),7.41 (m, 1H, H5), 7.44 (m, 2H, H5’, H20´), 7.47 (m, 1H, H10), 7.55 (m, 1H, H20), 7.56 (m, 2H, H9), 7.60 (m, 1H, H3), 7.61 (m, 2H, H9´), 7.64 (m, 1H, H6), 7.67 (m, 1H, H6’), 7.68 (m, 1H, H10´), 7.79 (m, 1H, H3´), 8.22 (m, 2H, H8’), 8.32 (m, 2H, H8) ppm. ^13^C NMR (125.78 MHz, CD_3_CN) δ 23.2 (d, ^3^*J*_PC_ 2.8 Hz, C12/C12’), 23.3 (d, ^3^*J*_PC_ 2.7 Hz, C12/C12’), 23.6 (d, 2C, ^3^*J*_PC_ 3.3 Hz, C13, C13’), 29.1 (C17), 29.2 (C17’), 29.3 (C18), 29.9 (C18’), 51.1 (d, 2C, ^2^*J*_PC_ 2.9 Hz, C11, C11’), 53.6 (C16), 53.7 (C16’), 73.4 (C15), 73.8 (C15’), 128.3 (d, 2C, ^3^*J*_PC_ 12.5 Hz, C5, C5’), 129.4 (d, ^1^*J*_PC_ 101.1 Hz, C7/C7’), 129.6 (d, ^1^*J*_PC_ 100.2 Hz, C7/C7’), 130.2 (d, ^3^*J*_PC_ 13.7 Hz, C9’), 130.4 (d, ^3^*J*_PC_ 13.8 Hz, C9), 133.72 (d, ^2^*J*_PC_ 11.2 Hz, C6), 133.69 (d, ^2^*J*_PC_ 10.8 Hz, C6’), 134.86 (d, ^4^*J*_PC_ 3.2 Hz, C10), 134.88 (d, ^2^*J*_PC_ 12.0 Hz, C8’), 135.02 (d, ^4^*J*_PC_ 2.8 Hz, C4’), 135.04 (d, ^4^*J*_PC_ 2.9 Hz, C4), 135.07 (d, ^2^*J*_PC_ 11.9 Hz, C8), 135.09 (d, ^4^*J*_PC_ 3.2 Hz, C10’), 136.6 (d, ^3^*J*_PC_ 16.5 Hz, C3), 137.1 (d, ^3^*J*_PC_ 16.0 Hz, C3’), 140.9 (d, ^2^*J*_PC_ 24.5 Hz, C2’), 142.1 (d, ^2^*J*_PC_ 24.5 Hz, C2), 142.5 (d, ^1^*J*_PC_ 121.8 Hz, C1), 142.8 (d, ^1^*J*_PC_ 120.8 Hz, C1’), 171.77 (C14/C14’), 171.8 (C14/C14’) ppm. ^31^P-NMR (67.48 MHz, CD_3_CN) δ ppm: 67.7, 68.2 ppm. ^15^N-NMR (50.67 MHz, CD_3_CN) δ 44.5 (d, ^1^*J*_NH19_ ≈ 70 Hz, N19), 45.1 (d, ^1^*J*_NH19’_ ≈ 69 Hz, N19’), 68.6 (N11), 69.5 (N11’) ppm. HRMS (ESI^+^) [M+H]^+^ calcd. for C_23_H_33_AuN_2_O_2_PS_2_, 661.1387; found, 661.1387. UV-Vis (λ_max_, nm [ε, 104 M^−1^·cm^−1^]): 235 (2.89), 325 (0.42)**.**

**Table S1**. ^31^P NMR (121.5 MHz) chemical shifts of [Au(dppta)Cl_2_] **2** under the different conditions used.

| **Conditions** | **δ_P_ (2)** | **Solvent** |
| --- | --- | --- |
| [Au(dppta)Cl_2_] | 69.8 | DMSO-*d*_6_ |
| [Au(dppta)Cl_2_] + culture medium ISO SENSITEST | 70.0 | DMSO-*d*_6_ |
| [Au(dppta)Cl_2_] + NaOH 0.1 N | 69.9 | DMSO-*d*_6_ |
| [Au(dppta)Cl_2_] + PBS | 68.8 | DMSO-*d*_6_ |
| [Au(dppta)Cl_2_] + HCl 0.1 N | 68.7 | DMSO-*d*_6_ |
| [Au(dppta)Cl_2_] + HCl 5 N | 69.2 | CD_3_OD |
| [Au(dppta)Cl_2_] + glycine | 68.9 | DMSO-*d*_6_ |
| [Au(dppta)Cl_2_] + ciprofloxacin | 68.0 | CD_3_CN |
| [Au(dppta)Cl_2_] + colistin | 68.1 | CD_3_CN |


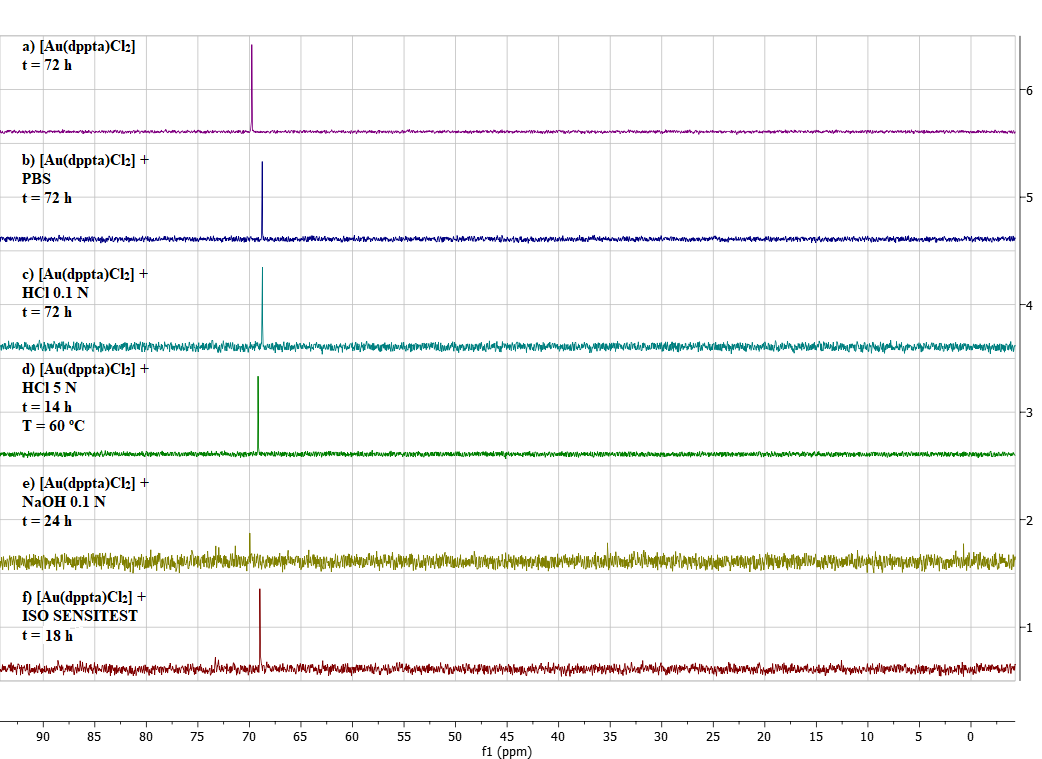


**Figure S1**. Comparison of ^31^P NMR spectra (121.5 MHz) of [Au(dppta)Cl_2_] complex **2**: a) in DMSO-*d*_6_; b) 0.4 mL DMSO-*d*_6_/0.1 mL of PBS; c) 0.4 mL DMSO-*d*_6_/0.1 mL of 0.1 N aqueous HCl; d) 5 M HCl in CD_3_OD; e) 0.4 mL DMSO-*d*_6_/0.4 mL of 0.1 N aqueous NaOH; f) 0.4 mL DMSO-*d*_6_/0.1 mL of ISO-SENSITEST culture medium.


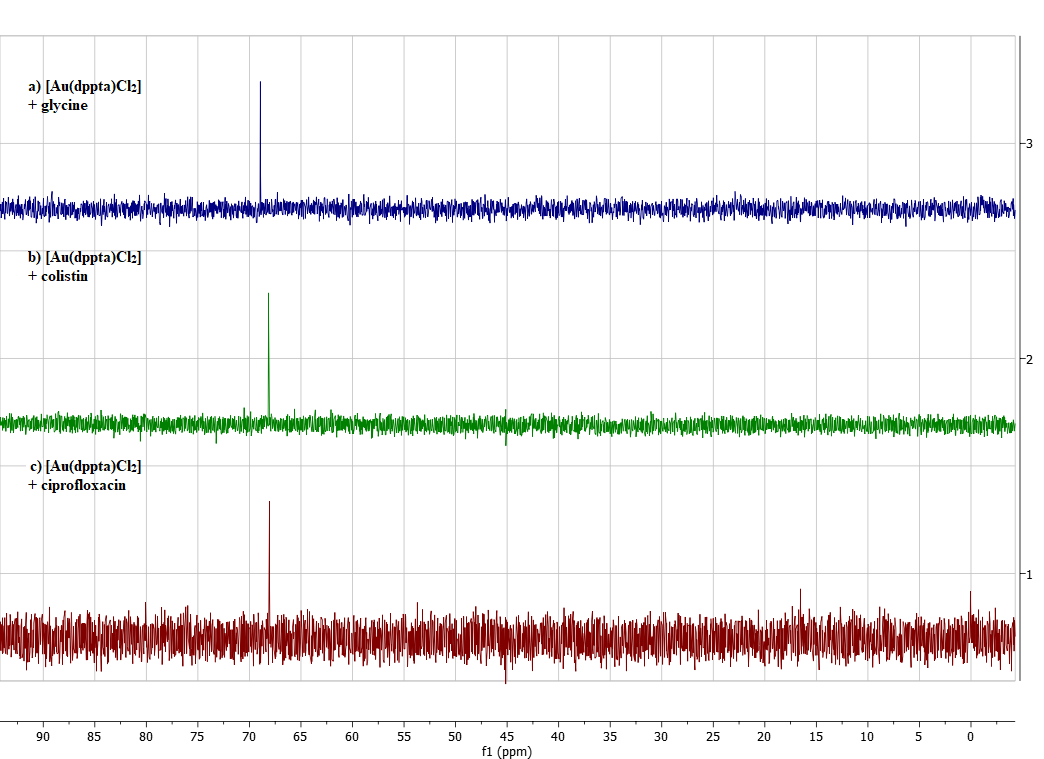


**Figure S2**. Comparison of ^31^P NMR spectra (121.5 MHz) of [Au(dppta)Cl_2_] complex **2**: a) in 0.4 mL DMSO-*d*_6_/0.1 mL of an equimolar amount of glycine in PBS; b) 0.25 mL CD_3_CN/0.2 mL of a 32 mg/mL aqueous solution of colistin/0.1 mL of culture medium; c) 0.25 mL CD_3_CN/0.25 mL of 40 mg/mL solution of ciprofloxacin in the culture medium/0.03 mL HCl 1 M.


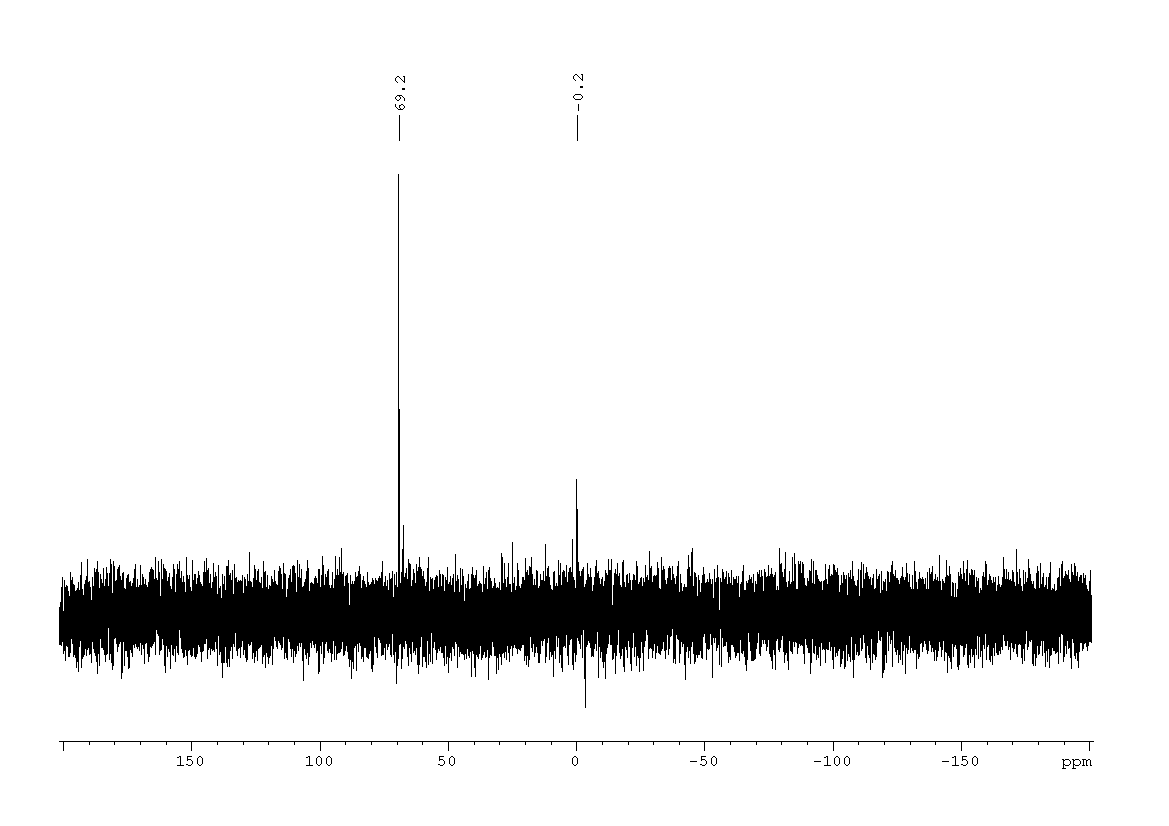


**Figure S3**. ^31^P NMR spectrum (242.95 MHz MHz) in DMSO-*d*_6_ of a lyophilisate resulting from the incubation of *A. baumannii* with complex **2** (8xMIC) during 1 h. The signal at δ -0.2 ppm arise from PBS.


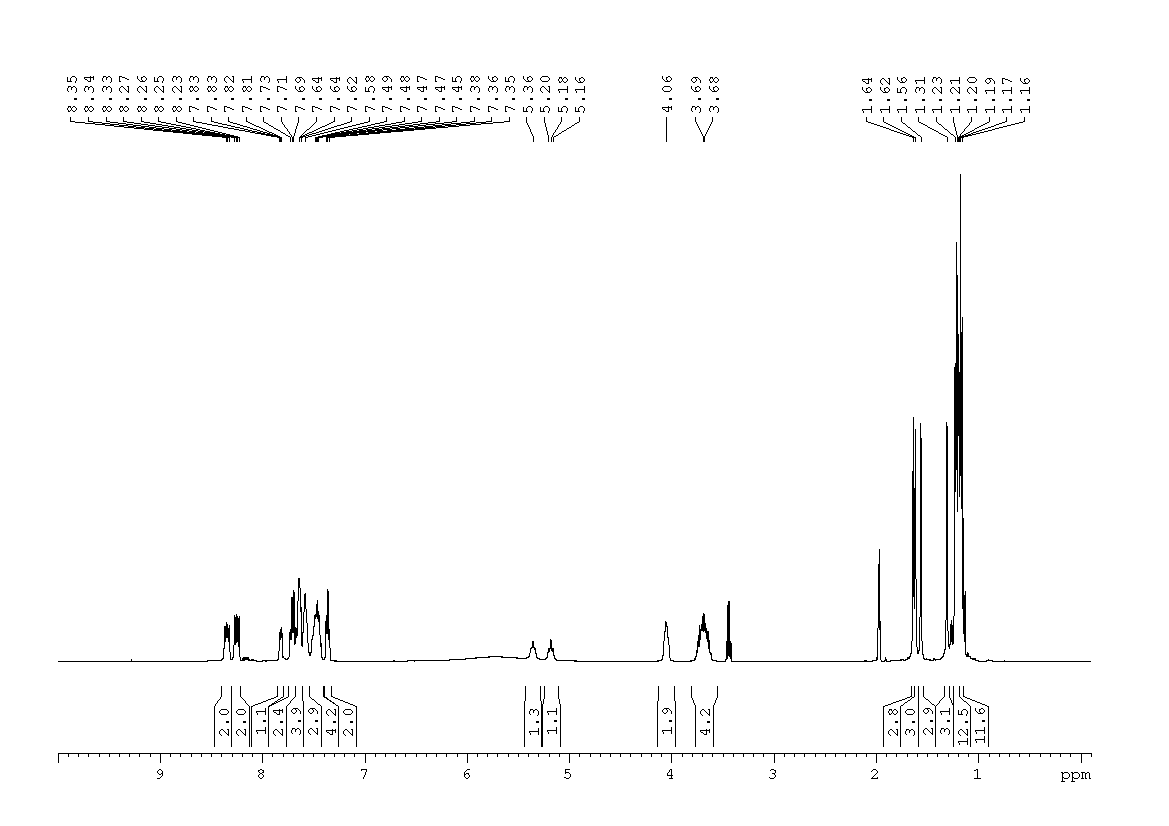


**Figure S4**. ^1^H NMR spectrum (500.13 MHz, CD_3_CN) of compound **3**.


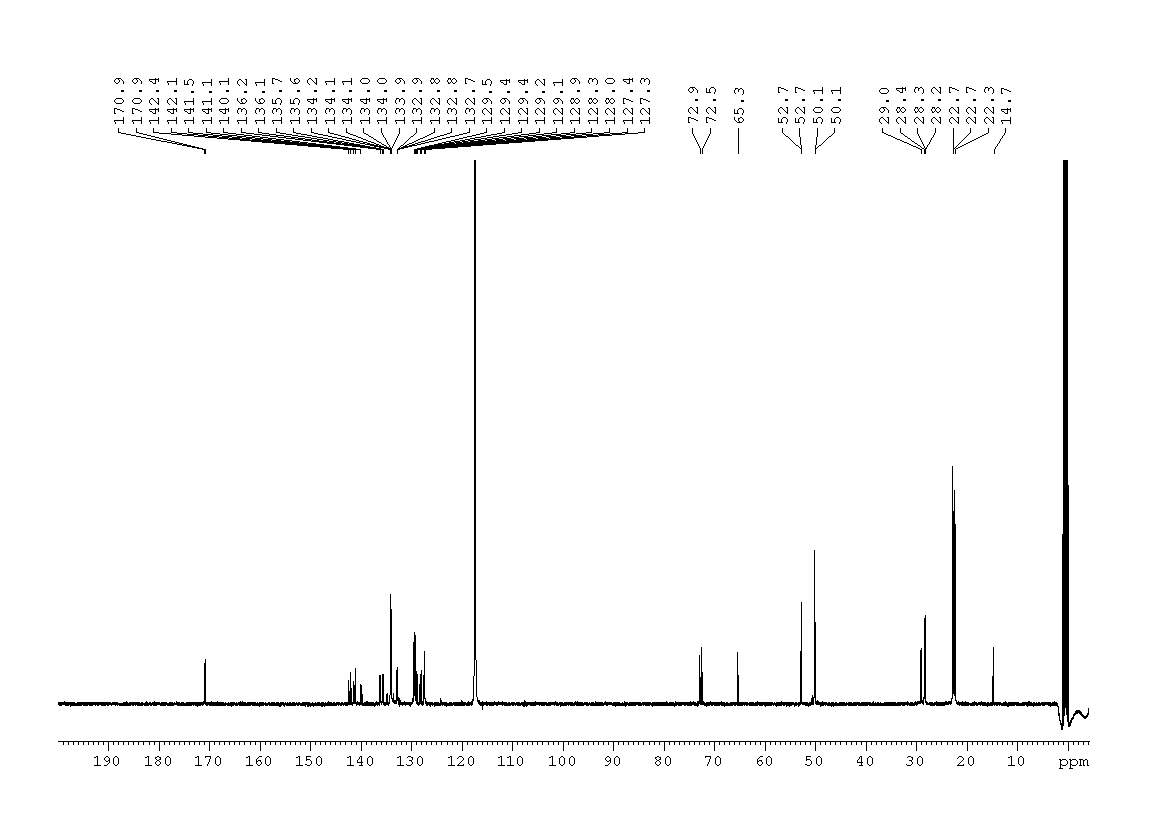


**Figure S5**. ^13^C NMR spectrum (125.76 MHz, CD_3_CN) of compound **3**.


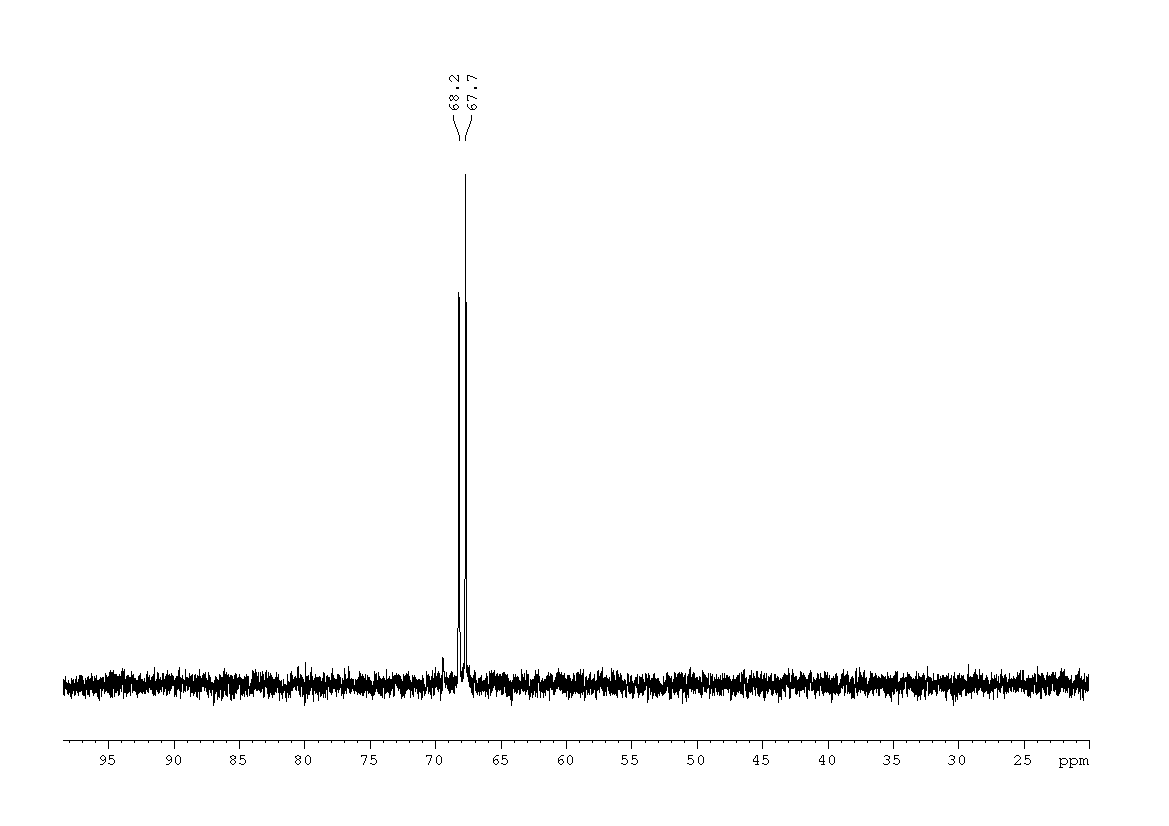


**Figure S6**. ^31^P NMR spectrum (202.46 MHz, CD_3_CN) of compound **3**.


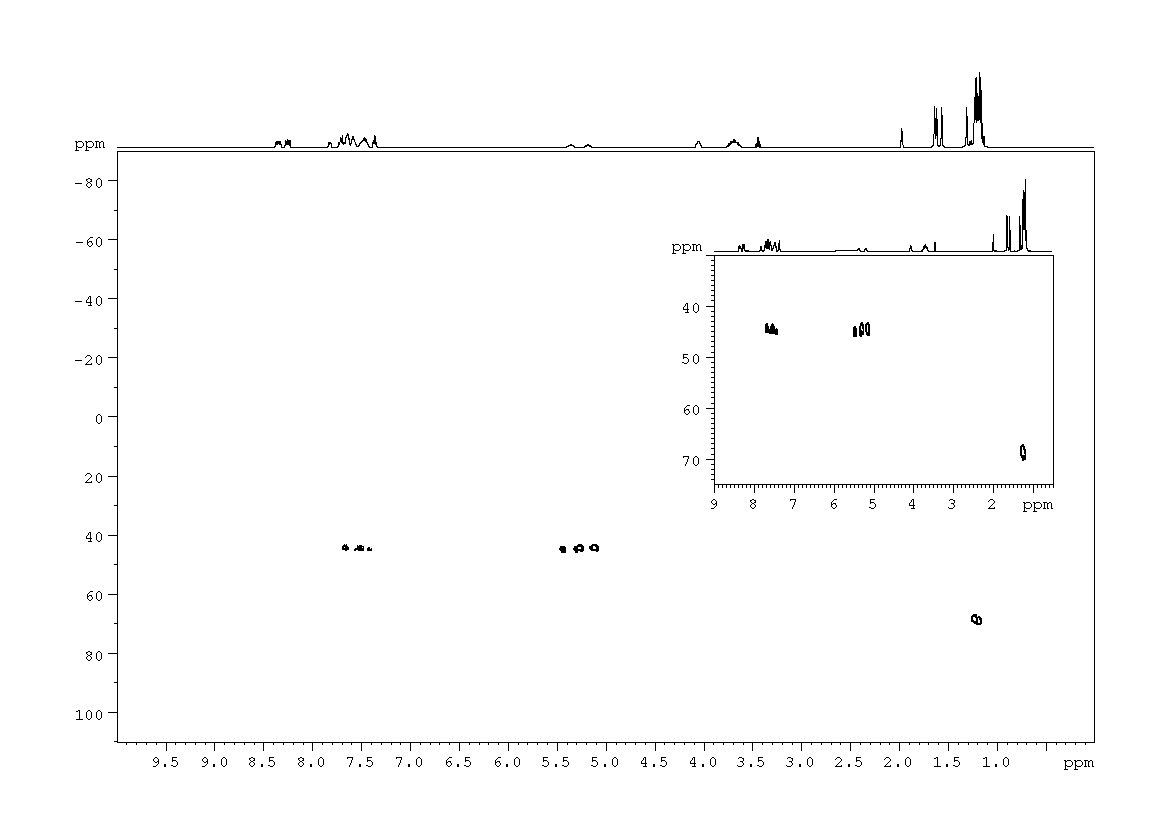


**Figure S7**. ^1^H,^15^N HMQC spectrum (500.13 MHz, CD_3_CN) of compound **3**.


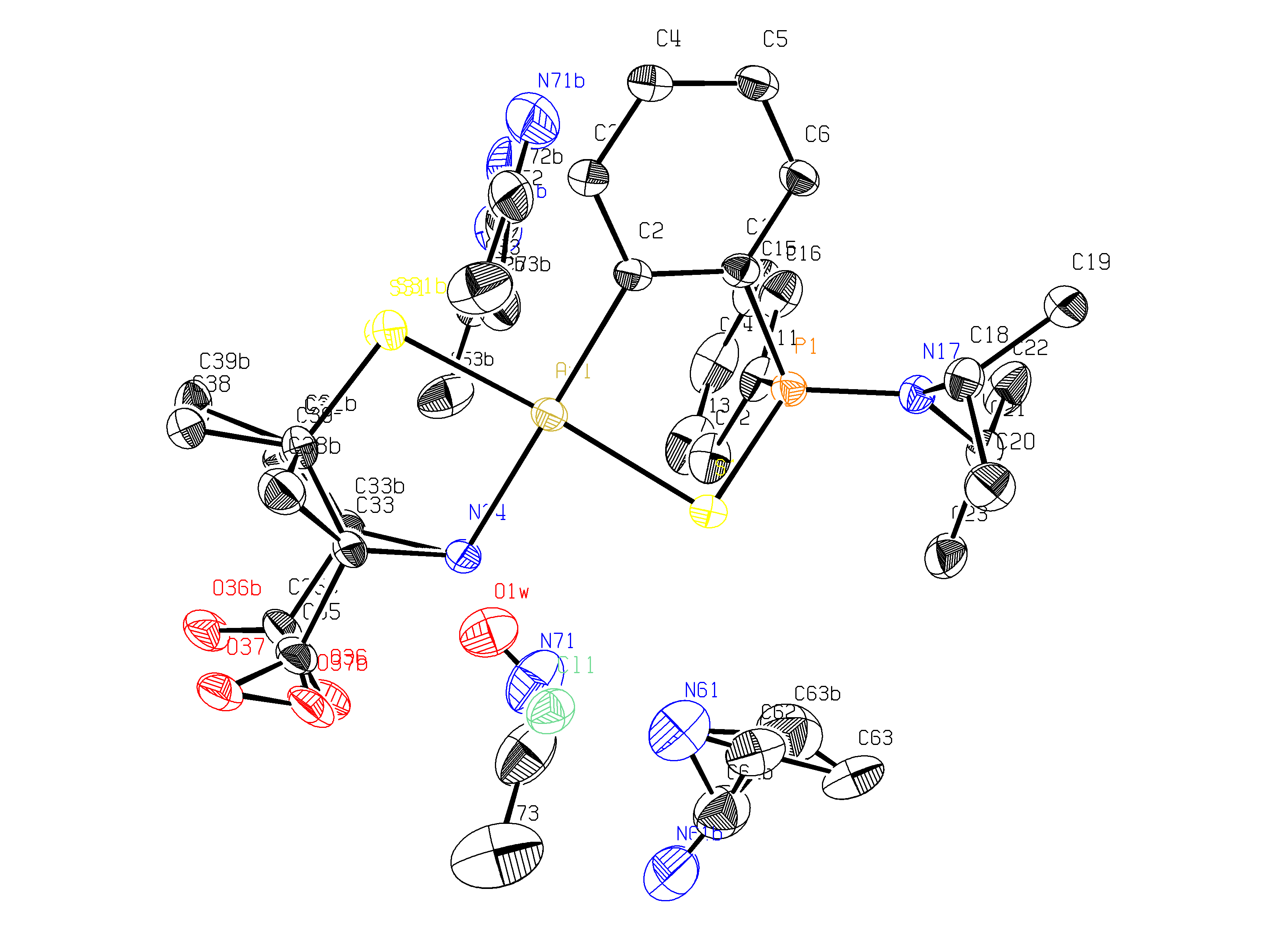


**Figure S8**. X-ray crystal structure of **3** (thermal ellipsoids shown at 30% probability) including atomic numbering. Hydrogen atoms have been omitted for clarity.

**Table S2**. Selected crystal data for compound **3**.

|  | **3** |
| --- | --- |
| **Empirical formula** | C_23_H_33_AuN_2_O_2_PS_2_·2.75(C_2_H_3_N)·Cl·0.5(O) |
| ***M*** | 817.92 |
| **Crystal System** | Triclinic |
| **Space group** | P1 |
| **Temperature/K** | 100 |
| ***a*/Å** | 11.4906 (8) |
| ***b*/Å** | 12.3205 (8) |
| ***c*/Å** | 14.1234 (9) |
| **α (°)** | 68.786 (6) |
| **β (°)** | 72.689 (6) |
| **γ (°)** | 88.495 (5) |
| ***V*/Å^3^** | 1772.1 (2) |
| ***Z*** | 2 |
| ***μ*/mm^-1^** | 10.29 |
| ***D_x_*/Mg cm^-3^** | 1.533 |
| **Crystal dimensions/mm** | 0.25 × 0.23 × 0.05 |
| ***F(000)*** | 817 |
| **θ range for data collection/°** | 3.5 – 69.3 |
| **Refls. measured** | 17237 |
| **Refls. unique** | 6546 |
| **Parameters/restraints** | 557/770 |
| ***R*1 [*I* ≥ 2σ(*I*)]** | 0.047 |
| ***wR*2 (all data)** | 0.118 |
| **Δρmax/Δρmin/e⋅Å^-3^** | 2.22/-2.01 |

**References**

Belmonte-Sánchez, E., Iglesias, M. J., El Hajjouji, H., Roces, L., García-Granda, S., Villuendas, P., Urriolabeitia, E. P., López-Ortiz F. (2017). Cycloaurated phosphinothioic amide complex as a precursor of gold(I) nanoparticles: efficient catalysts for A3 synthesis of propargylamines under solvent-free conditions. Organometallics. 36, 1962−1973. doi: 10.1021/acs.organomet.7b00102.

Bruker, APEX3 v2014.5-0 (2014). Bruker AXS Inc., Madison, Wisconsin, USA.

Bruker, SAINT V8.38A (2017). Bruker AXS Inc., Madison, Wisconsin, USA.

Bruker, SADABS (2016). Bruker AXS Inc., Madison, Wisconsin, USA.

Sheldrick, G. M. (2008). A short history of SHLEX. Acta Cryst. A 64, 112-122. doi: 10.1107/S0108767307043930

Farrugia, L. J. (2012). WinGX and ORTEP for Windows: an update. J. Appl. Crystallogr. 45, 849-854. doi: 10.1107/S0021889812029111.
